# The regulatory landscape of a core maize domestication module controlling bud dormancy and growth repression

**DOI:** 10.1038/s41467-019-11774-w

**Published:** 2019-08-23

**Authors:** Zhaobin Dong, Yuguo Xiao, Rajanikanth Govindarajulu, Regina Feil, Muriel L. Siddoway, Torrey Nielsen, John E. Lunn, Jennifer Hawkins, Clinton Whipple, George Chuck

**Affiliations:** 1 Plant Gene Expression Center/USDA, University of California, Berkeley, Albany, CA 94710 USA; 2 Brigham Young University, Provo, UT 84602 USA; 3 West Virginia University, Morgantown, WV 26506 USA; 4 Max Planck Institute of Molecular Plant Physiology, Muehlenberg, 14476 Potsdam-Golm, Germany

**Keywords:** Plant development, Patterning, Plant domestication

## Abstract

Many domesticated crop plants have been bred for increased apical dominance, displaying greatly reduced axillary branching compared to their wild ancestors. In maize, this was achieved through selection for a gain-of-function allele of the TCP transcription factor *teosinte branched1* (*tb1*). The mechanism for how a dominant *Tb1* allele increased apical dominance, is unknown. Through ChIP seq, RNA seq, hormone and sugar measurements on 1 mm axillary bud tissue, we identify the genetic pathways putatively regulated by TB1. These include pathways regulating phytohormones such as gibberellins, abscisic acid and jasmonic acid, but surprisingly, not auxin. In addition, metabolites involved in sugar sensing such as trehalose 6-phosphate were increased. This suggests that TB1 induces bud suppression through the production of inhibitory phytohormones and by reducing sugar levels and energy balance. Interestingly, TB1 also putatively targets several other domestication loci, including *teosinte glume architecture1*, *prol1.1/grassy tillers1*, as well as itself. This places *tb1* on top of the domestication hierarchy, demonstrating its critical importance during the domestication of maize from teosinte.

## Abstract (web summary)

The TB1 transcription factor was selected for the increased apical dominance of maize compared to its ancestor teosinte. A metabolic and genomic analysis of domesticated axillary buds suggest that TB1 achieved this by regulating phytohormone signaling, sugar metabolism and other domestication genes.

## Introduction

A series of regulated branching decisions made during development is critical in determining plant architecture. During vegetative growth the shoot apical meristem produces successive leaves with vegetative branches initiating as lateral meristems in their axils. These branches, called tillers in grasses when formed at ground level, are in turn capable of reiterating growth of the main shoot and initiate branches themselves. Without any mechanism to repress the growth of these structures, plants would branch continuously at great cost. Thus, optimal growth can only be achieved by regulating branching over the course of normal development in response to ideal environmental conditions and proper energy status.

Modification of branching architecture has adaptive consequences, and was an important factor in the domestication of the unbranched maize plant (*Zea mays* ssp. *mays*) from its highly tillered wild ancestor, teosinte (*Z. mays* ssp. *parviglumis*)^1^. Like many angiosperms, maize axillary meristems are initiated by an auxin-dependent process that also requires the local activity of several transcription factors^2^. However, suppression of tiller growth in domesticated crops such as maize is not regulated at the axillary meristem initiation stage. Instead, this process occurs after the axillary meristem has already formed a few protective leaves. Shortly thereafter, growth of the bud arrests, and enters a state of dormancy. The decision to either establish or break this dormancy is tightly regulated by a complex interaction between internal as well as external stimuli.

Whole plant, systemic transport of phytohormones is known to be critical for regulation of bud growth^3^. A classical example is the phenomenon of apical dominance, in which the meristem of the growing apex produces a long distance inhibitory cue that maintains axillary buds in a dormant state. Although the precise mechanism of apical dominance remains unclear^3^, it appears to involve the transport of auxin downward from its source in the growing apex, along with the upward transport of cytokinin and strigolactones^4–6^. A particular difficulty in understanding the mechanism by which long distance transport of a phytohormone like auxin can regulate bud dormancy came from the observation that basally transported auxin fails to enter dormant buds^7,8^, requiring some unknown local mechanism within axillary buds to regulate dormancy. An intriguing recent study in pea^9^, confirmed in other systems^10^, demonstrated that redistribution and transport of sugars (photosynthates) into growing buds provides an important cue to break dormancy caused by apical dominance. This is consistent with a growing body of data suggesting that sugars comprise an important class of signaling molecules that regulate diverse plant developmental processes^11^. Thus, the interaction of several classes of transported phytohormones, as well as sugar sensing within the plant are implicated in the regulation of bud dormancy.

While it is not clear how these diverse signals are integrated, increasing evidence suggests that transcription factors expressed within the initiating bud act downstream of these cues as master regulators of bud dormancy. For example, the TCP transcription factor *teosinte branched1* (*tb1*) is a key regulator of apical dominance and tiller bud dormancy in maize^12^. *tb1* mutants overproduce tillers and aerial branches, indicating that the gene functions as a repressor of bud growth in multiple developmental contexts. *tb1* orthologs in eudicots and other grasses have a similar role in bud dormancy and branching^13–15^. In maize, *tb1* regulates aerial branching by targeting genes that alter branch fates, such as the *BTB POZ* domain gene *tassels replace upper ears1* (*tru1*)^16^. In addition, the class I HD-ZIP transcription factor *grassy tillers1* (*gt1*), may function downstream of *tb1* to control tillering at ground level because *tb1* is required for *gt1* to be expressed^17^. Interestingly, *tb1* and *gt1* levels are sensitive to FR/R ratios as well as other environmental and hormonal branching cues in monocots^13,17,18^, suggesting that together they comprise a regulatory hub that controls bud dormancy in response to diverse internal and external branching signals. Their importance as integrators of bud dormancy signals is further underscored by the fact that regulatory variants at both loci have been selected during maize domestication, leading to the reduced branching found in maize compared to teosinte^19,20^. Taken together, these studies demonstrate that *tb1* and *gt1* are at key regulatory positions, integrating multiple cues that switch between dormancy and growth in the bud.

Despite the importance of *tb1* and *gt1* in the regulation of bud dormancy, little is known about the factors they control to promote this process. Recent work in *Arabidopsis* has shown that abscisic acid (ABA) production and signaling is directly regulated by orthologs of *tb1* and *gt1*^21^, consistent with the known roles of ABA in dormancy of buds^22^. It is still unclear how *tb1* and *gt1* integrate ABA signaling with the other phytohormones, sugars, and other environmental signals known to affect bud growth. Additional work in maize has shown that TB1 directly regulates transcription factors with known developmental roles^16,23^. Two of these genes, *teosinte glume architecture* (*tga1*) and *tru1*, have also been implicated in maize domestication, leading to the hypothesis that *tb1* may in fact control a domestication regulatory network^23,24^.

To better understand the regulatory network of bud dormancy under the control of *tb1* and *gt1*, we have profiled transcriptional changes associated with *tb1–gt1-*mediated bud dormancy in maize tiller buds and identified putative direct targets bound by TB1. We show that TB1 putative direct targets include *gt1*, and that together they regulate a complex of plant signaling networks including the phytohormones ABA and jasmonic acid (JA) in addition to sugar signaling, consistent with direct hormone and sugar metabolite measurements. In addition, we provide evidence that TB1 directly binds to regulatory regions in its own promoter, as well as in the promoters of the domestication loci *prol1.1* and *tga1*. These results help explain how allelic variation in the *tb1* regulatory hub during domestication was able to produce such drastic, beneficial agronomic changes.

## Results

### *tb1* and *gt1* tiller buds fail to establish dormancy

**Fig. 1:**
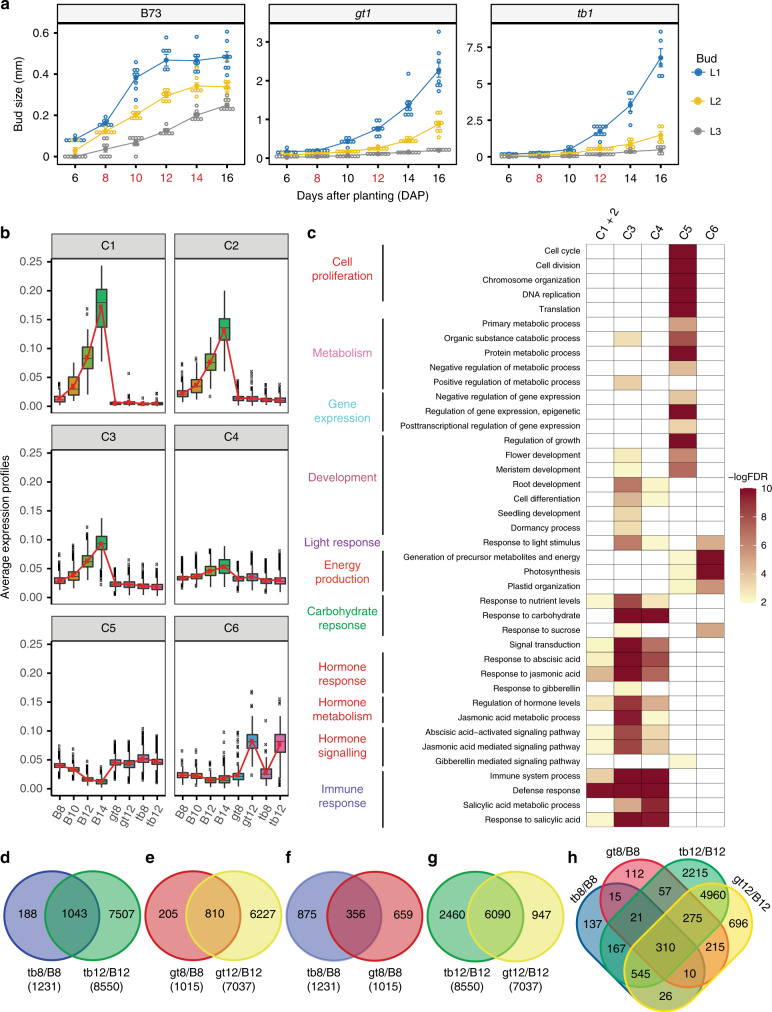
Transcriptional profiling of bud dormancy regulated by *gt1* and *tb1*. **a** Length of tiller buds in the first (L1), second (L2), and third (L3) leaf axis of B73, *gt1* and *tb1* at 6, 8, 10, 12, 14, and 16 days after planting (DAP). Buds from development stages in red on the *x*-axis were collected for RNA-seq transcriptome profiling. Bars for each stage represent the mean ± standard error of eight replicates. **b** Six co-expression clusters (C1–C6) were identified from the 6998 genes that were differentially expressed across the B73 developmental series, *gt1* and *tb1*. Clusters have been sorted such that those with similar mean vectors (as measured by the Euclidean distance) are plotted next to one another. Connected red lines correspond to the mean expression profiles for each cluster. The vertical bars define the upper or lower quartile, and dots outside the bars indicate outliers. **c** Over-represented GO terms in the co-expression clusters identified in **b**. Go terms with false discovery rate (FDR) ≤ 0.01 (−logFDR ≥ 2) were considered as significantly enriched. **d**–**h** Venn diagrams showing common or uniquely differentially expressed genes between early (pre-dormancy at 8 DAP) and late (post-dormancy at 12 DAP) stage in *tb1* and *gt1* compared to B73. B8, B10, B12, and B14 represent B73 tiller buds at 8, 10, 12, and 14 DAP, respectively; similarly tb8, tb12, gt8, and gt12 stand for *tb1* and *gt1* tiller buds at 8 and 12 DAP, respectively

To understand the growth dynamics associated with maize tiller bud dormancy and expansion in wild type (B73), *tb1* and *gt1*, we monitored tiller bud growth in the first (L1), second (L2), and third (L3) leaf axils from 6 to 16 days after planting (DAP). As shown in Fig. 1a, the L1 tiller buds in B73 grew rapidly from 6 to 10 DAP, with a notable reduction in growth rate from 10 to 12 DAP and complete cessation of growth after 12 DAP. Similar growth dynamics were detected in B73 L2 and L3 tiller buds which were developmentally delayed compared to L1 buds, suggesting that progression to dormancy follows a regular developmental sequence that is autonomous to each bud. In contrast, tiller buds of *tb1* and *gt1* mutants grew rapidly without obvious inhibition up to 16 DAP (Fig.1a). The growth rate of *tb1* buds was significantly larger than *gt1* buds, suggesting that while both *tb1* and *gt1* are necessary to initiate dormancy, they differentially regulate the rate of bud expansion. Thus, under our controlled growth conditions, dormancy of B73 tiller buds is established by *tb1/gt1* over a 4-day window from 8 to 12 DAP.

### Transcriptional dynamics of tiller buds

These bud measurements provide useful reference points to investigate the transcriptional dynamics associated with the onset of dormancy. We chose to generate RNA-seq transcriptomes from L1 tiller buds, and at stages that would provide the most insight into the onset of dormancy. For B73 buds, we chose four developmental time points (8, 10, 12, and 14 DAP) that span the bud stages from pre- to post-dormancy. We chose two stages for continuously growing *tb1* and *gt1* buds (8 and 12 DAP) correlating with clear pre- and post-dormancy stages in wild type (Fig. 1a and Supplementary Fig. 1). We used several methods to validate the reliability of the resulting transcript profiles. Principal component and hierarchical clustering analyses confirmed that the biological replicates were highly correlated as were developmental stages within and across genotypes (Supplementary Figs. 2 and 3). A spot check of *tb1* and *gt1* transcript levels corroborated previous observations^17^ that both *tb1* and *gt1* were downregulated in *tb1* mutants, while *tb1* levels were not changed in *gt1* mutants (Supplementary Table 1), supporting the model in which *tb1* is upstream of *gt1*. In addition, *gt1* transcripts from the *gt1* mutant were consistent with the predicted splice donor site mutation of this allele (Supplementary Fig. 4). Together, these analyses indicate that we generated a reliable set of dormancy-related transcriptomes that will provide insight into the roles of *tb1* and *gt1* in the regulation of tiller outgrowth in maize.

In total, 23,343 genes were expressed in at least one sample (Supplementary Data 1 and 2), of which 40% (9388) were differentially regulated across the dormancy series in B73 (Supplementary Data 3). A similar proportion (42%, 9761) were differentially expressed between B73 and the two tillering mutants (Supplementary Data 4), but a slightly smaller set of these (30%, 6998) were differentially expressed across both the developmental series and between B73 and the two tillering mutants (Supplementary Data 5), most likely reflecting genes that regulate the normal progression of dormancy downstream of *tb1* and *gt1*. *K*-Means clustering^25^ of this subset identified six gene co-expression clusters (Supplementary Data 6). Clusters 1–4 showed a similar pattern of increasing expression during dormancy acquisition in B73 tiller buds, but consistently low expression in *gt1* and *tb1*, likely containing genes that promote bud dormancy, some of which may be activated by *tb1* and *gt1* (Fig.1b). In contrast, cluster 5 genes showed the opposite pattern, and likely include genes that are associated with bud dormancy, some of which may be down-regulated by *tb1* and *gt1*. Genes in cluster 6 show increased expression only in late stage *gt1* and *tb1* buds, containing genes likely associated with rapid growth and expansion at later stages when dormancy is bypassed. Gene ontology (GO) enrichment analysis identified several distinct physiological processes associated with these clusters (Fig. 1c). Overall, clusters 1–4 are enriched for genes involved in responses to carbohydrates, hormone metabolism and signaling, and immune response. In particular, genes involved in metabolism and signaling of ABA, JA, and gibberellic acid (GA) are enriched in clusters 1–4, indicating an important role for these hormones in the regulation of maize bud dormancy. Clusters 3 and 4 are also enriched for genes involved in light stimulus and plant development. Cluster 5 is enriched for genes that participate in cell proliferation, metabolism, gene expression regulation, photosynthesis, and GA signaling. Cluster 6 is enriched for genes involved in response to light stimulus, synthesis of metabolic precursors, energy status, photosynthesis, and response to sucrose. This suggests that tiller buds may require a sustainable energy supply during later stages of active tiller bud outgrowth in the *tb1* and *gt1* mutants. Collectively, these data show that *tb1* and *gt1* are required to establish bud dormancy, possibly through regulation of hormone metabolism/signaling, carbohydrate response, light response/photosynthesis, cell proliferation, and core metabolic processes.

There is a substantial (~7-fold) increase in the number of differentially expressed genes (DEGs) from early (pre-dormancy) to late (post-dormancy) B73 buds compared to both *tb1* and *gt1* at similar stages, likely reflecting the significant physiological differences of rapidly growing versus dormant buds. However, for both mutants the majority (80–85%) of the genes differentially expressed at pre-dormancy, were also differentially expressed later, indicating that a core set of genes is regulated at both stages (Fig. 1d and e, Supplementary Data 7–12). Based on the similar mutant phenotypes, and the evidence that *gt1* expression requires *tb1* activity, we expected most genes differentially regulated in *gt1* to be similarly regulated in *tb1*. Surprisingly, only 35% of the early stage *gt1* DE transcripts were also differentially expressed in *tb1*. This situation was reversed at the later stage where most (86%) of the genes differentially expressed in *gt1* were also differentially expressed in *tb1* (Fig. 1f–h, Supplementary Data 13–15).

### Identification of potential TB1 targets

**Fig. 2:**
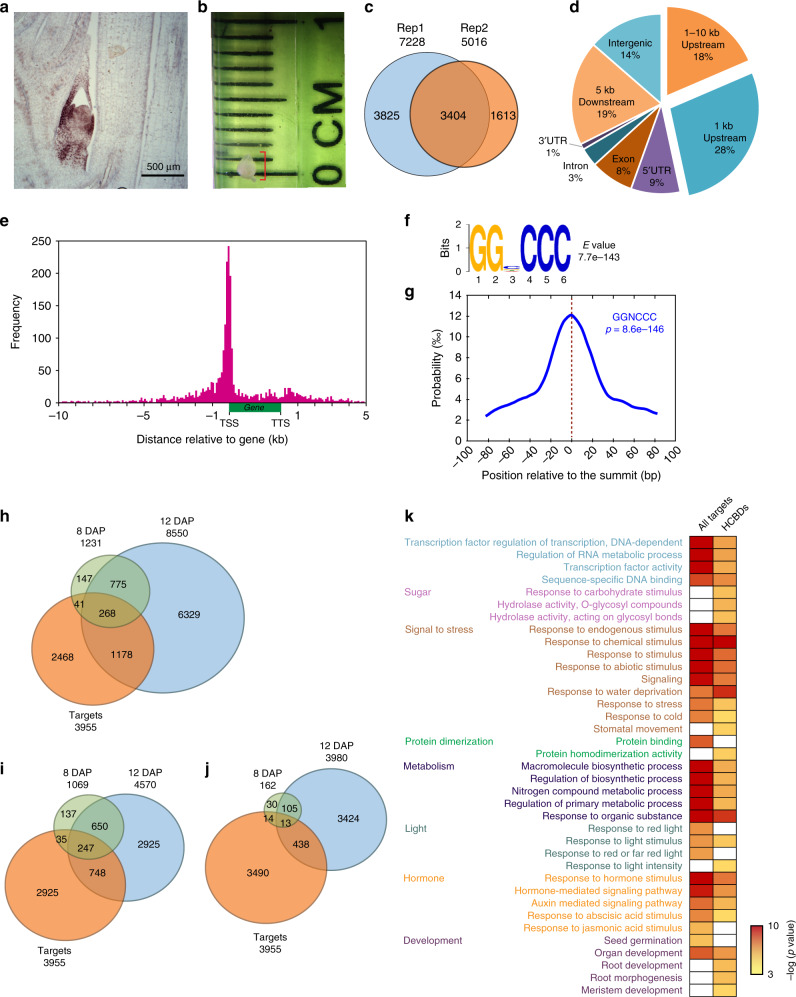
Genome-wide binding profile for TB1. **a** TB1 Immunolocalization on developing axillary bud from 3-week-old B73 seedling. **b** Size of the axillary buds used for ChIP. **c** Overlap between the two ChIP seq replicates relative to the IgG controls. **d** Genome-wide distribution of TB1-binding peaks. **e** Distribution of TB1-binding peaks relative to gene models showed strong enrichment within 1 kb upstream of the transcription start sites (TSS). **f** Enrichment of GGNCCC motifs within the TB1-binding peaks. **g** Localization of the GGNCCC motif relative to TB1 peak summits. **h** Overlap of all TB1 ChIP targets and all *tb1* differentially expressed genes. **i** Overlap of all TB1 ChIP targets and *tb1* down-regulated differentially expressed genes. **j** Overlap of all TB1 ChIP targets and *tb1* up-regulated differentially expressed genes. **k** Functional categories of all the TB1 targets and the 268 high confidence bound DEGs (HCBDs) identified in **h**

A specific antibody was generated to the C-terminus of the maize TB1 protein outside the TCP domain^16^. When used for immunolocalization on young shoot tissue, the antibody localizes to the nuclei of early initiating axillary meristems, as well as older buds, including several leaf primordia and the unexpanded stem (Fig. 2a). In our corn field, arrested B73 buds are ~1 mm long including the surrounding leaves (Fig. 2b). To perform a ChIP-seq experiment, 3–4 arrested buds were harvested from 2.5 to 3-week old field-grown B73 plants. A total of 20 g of bud tissue was isolated from several thousand plants, an amount enough for 10 ChIP experiments (see the “Methods” section). The ChIP DNAs were tested for enrichment of a known TB1 direct target, *tru1*, as well as a putative direct target, *gt1*, before pooling the DNAs from five ChIP experiments as single biological replicates, allowing the construction of two ChIP sequencing libraries. As a negative control, chromatin bound to IgG was isolated using previously described protocols^26^. The specific tissue dissection, large amount of starting material, and multiple replicates all contributed to the specific binding and low background noise detected in the resulting ChIP-seq analysis.

The overlap of the ChIP-seq data between the two biological replicates relative to the IgG controls identified 3404 reproducible peaks (Fig. 2c, see the “Methods” section). Only the reproducible peaks were used for the following analysis, and the majority of them (86%) map to genic regions, with only 14% mapping to intergenic regions (Fig. 2d). Analysis of the genic peaks indicates that TB1 binds preferentially to promoters (Fig. 2d and e). A total of 46% of the peaks map within 10 kb upstream of the transcription start site (TSS), and over 60% of these promoter peaks localized within 1 kb upstream of the TSS (Fig. 2d and e). A significant number of peaks, however, map to other regions, including downstream and intergenic regions. Surprisingly, very few peaks map to 3′ UTRs or introns, despite the fact that TB1 is capable of binding to the latter^16^.

TB1 is categorized as a class II TCP protein, known to bind the consensus sequence GTGGNCCC^27^. To determine if a similar sequence exists among the TB1-bound regions, a motif search was performed amongst the peak sequences. The most frequently found motif was GGNCCC positioned directly within the peak summits (Fig. 2f and g), supporting the previously identified consensus sequence.

We correlated the reproducible peaks with corresponding target genes by requiring the peaks to map within 10 kb upstream to 5 kb downstream of the gene, which resulted in 3955 genes as potential TB1 targets (Supplementary Data 16). To determine which of these are transcriptionally modulated by TB1 in our experimental conditions, we correlated the TB1 ChIP target set with *tb1* DEGs, and found 309 and 1446 genes were differentially expressed at 8 DAP and 12 DAP, respectively (Fig. 2h). Among them, 268 genes (designated as high-confidence bound DEGs, HCBDs) were modulated at both time points, including 247 downregulated genes versus 13 upregulated in *tb1* mutants (Fig. 2i and j), indicating that TB1 may function primarily as a transcriptional activator. Functional GO analysis revealed all targets, including HCDBs, could be classified within the following categories: transcription factors, signaling response to stress, biosynthetic metabolism, or phytohormone pathways (Fig. 2k). In addition, these target datasets showed enrichment in the GO category of light response, including R/FR response (Fig. 2k), supporting previous findings that the *tb1-gt1* module is part of the shade avoidance response downstream of PHYB^17,18^.

### TB1 regulates phytohormones

Previous work suggests that *tb1* and *gt1* orthologs in *Arabidopsis* may promote dormancy by directly modulating ABA levels^21^. Our transcript profiling similarly implicates ABA in maize bud dormancy, but we also found evidence for the involvement of additional hormones, such as JA, GA, and auxin/indole-3-acetic acid (IAA) (Figs. 1c and 2k). Given the critical importance of these hormones in diverse plant developmental processes, we chose to carefully investigate their biosynthesis, metabolism, and abundance relative to *tb1* and *gt1*.

**Fig. 3:**
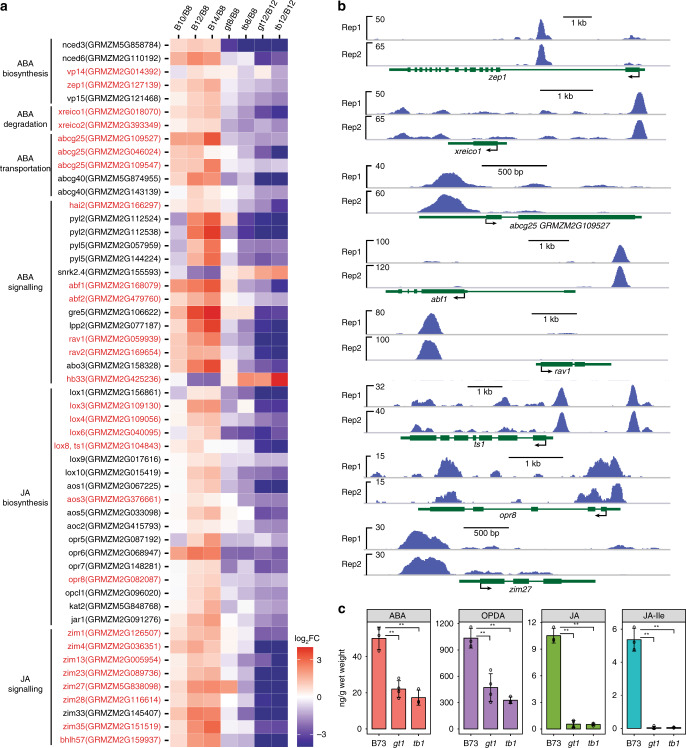
Bud dormancy is associated with *tb1*-*gt1-*mediated regulation of ABA and JA hormone homeostasis and signaling. **a** Genes involved in ABA biosynthesis, ABA degradation, ABA transportation, ABA signaling, JA biosynthesis and JA signaling were up-regulated in tiller buds across the B73 developmental series (B10/B8, B12/B8, B14/B8), but down-regulated in growing *gt1* and *tb1* buds. Stages and genotypes are indicated as described in Fig. 1, with slashes representing pairwise comparison between two samples (e.g. B10/B8 is a pairwise comparison between B73 buds at 10 vs. 8 DAP). Genes were highlighted in red if they were also putative direct targets of TB1 identified by ChIP-seq. FC fold change. Gradient color scale indicates the log value of expression fold change (log_2_FC). **b** TB1 ChIP-Seq-binding peaks near differentially expressed ABA and JA genes from (A). rep1 and rep2 represent two biological replicates of the TB1 ChIP-seq assay. **c** Quantification of ABA, OPDA, JA, and JA-Ile levels in tiller buds of B73, *gt1*, and *tb1* at 12 DAP. OPDA 12-oxo-phytodienoic acid, JA-Ile Jasmonic acid-isoleucine. Data are means ± SE calculated from at least three biological replicates. **p < 0.01; two-tailed Student’s *t-*test

ABA metabolic genes, including the *NINE-CIS-EPOXYCAROTENOID DIOXYGENASE*s (*NCEDs*) were up-regulated during the progression to dormancy in B73 buds, but down-regulated in *tb1* and *gt1* (Fig. 3a). A similar pattern was observed for the orthologs of ABA transporter genes (*ABCG*25 and *40*)^28,29^, and for *XERICO* which is known to inhibit ABA degradation (Fig. 3a)^30^. ABA synthesis may be directly regulated by TB1 as our ChIP-seq showed TB1 binding to the promoters of maize ABA biosynthetic genes *zep1* and *vp14*, ABA degradation inhibitors *xerico1* and *2*, as well as the orthologs of the ABA transporter *ABCG25* (Fig. 3b). To correlate the functions of these genes to endogenous ABA levels in dormant buds, we compared ABA levels in buds of dormant B73 vs. growing *tb1* and *gt1*. We found a significant reduction of ABA in buds of *tb1* and *gt1* mutants, confirming that both genes promote ABA accumulation during dormancy (Fig. 3c). As expected, the reduction in ABA levels corresponded with significant expression changes in the downstream ABA gene regulatory network in *tb1* and *gt1* (Fig. 3a). Several of these network genes were also bound by TB1, including maize orthologs of *HIGHLY ABA-INDUCED PROTEIN PHOSPHATASE 2C* (*HAI1*), *ABA RESPONSIVE ELEMENT BINDING FACTOR1* and *2* (*ABF1, 2*)*, RELATED TO ABI/VP1 1* and *2* (*RAV1, 2*), and *HOMEOBOX PROTEIN33* (*HB33*) (Fig. 3a, b). Thus, *tb1* and *gt1* not only promote ABA synthesis and transport, but may also directly modulate ABA signal transduction to maintain bud dormancy.

Unlike ABA, JA was not implicated in promoting axillary bud dormancy until only recently^31,32^ Surprisingly, a recent report indicated that JA may promote bud growth^33^. However, our transcript profiling showed strong evidence that both JA biosynthesis and downstream signaling are affected by *tb1/gt1-*mediated bud dormancy (Fig. 3a). Orthologs of known JA biosynthesis genes including *LIPOXYGENASE*s (*LOX*s), *OXOPHYTODIENOATE REDUCTASEs* (*OPR*s), and others were down-regulated in both *tb1* and *gt1*, but up-regulated during the progression to dormancy in B73. Several of these were also putatively bound by TB1 (Fig. 3a, b), including *OPR8* which is necessary for JA production and inhibition of reproductive branch growth in maize^34^, as well as *tasselseed1* (*ts1*), a JA biosynthetic gene necessary for male sexual identity in the tassel spikelets^35^ (Fig. 3b). To validate the association of *tb1* and *gt1* with JA, we directly measured levels of JA, the JA biosynthetic intermediate 12-oxo-phytodienoic acid (OPDA), as well as the bioactive form of JA, jasmonyl-isoleucine (JA-Ile), and found all were decreased in *tb1* and *gt1* mutant buds. Specifically, there was a striking decrease of both JA and JA-Ile, providing strong evidence that *tb1* and *gt1* are necessary for the accumulation of bioactive JA in dormant buds (Fig. 3c). Downstream JA signaling components, including members of the *JASMONATE-ZIM DOMAIN PROTEIN*s (*JAZ*) transcription factor family, *basic HELIX-LOOP-HELIX57* (*bHLH57*), and homologs of *JASMONATE-ASSOCIATED MYC2-LIKE* (*JAM*), were differentially regulated in *tb1* and *gt1* and bound by TB1 (Fig. 3a, b). This suggests that *tb1* and *gt1* are not only important for JA production in axillary buds, but also fine tune downstream JA signaling during dormancy.

We detected differential regulation of other hormone signaling networks in addition to JA and ABA in our transcript profiling, especially GA and auxin. GA is known to associate with actively growing and elongating tissues^36,37^. We found evidence of regulation of GA biosynthesis, GA inactivation and increased GA signaling in growing *tb1* and *gt1* buds, suggesting that *tb1*/*gt1* may inhibit GA production and signaling to promote bud dormancy (Supplementary Fig. 5). Auxin signaling was also differentially regulated, with several known auxin-responsive genes being both up-regulated or down-regulated and bound by TB1 (Supplementary Fig. 6). However, we found no physiological evidence that auxin biosynthesis was regulated by *tb1/gt1*, since IAA measurements showed no significant difference between dormant B73 and growing *tb1* and *gt1* buds (Supplementary Fig. 6A). In addition, no auxin biosynthetic or transport genes were found among putative direct targets of TB1, suggesting auxin homeostasis may not be the major target of *tb1*/*gt1* to control tiller outgrowth, although this does not rule out the possibility that auxin mediates apical dominance either upstream of, or through genes outside of the *tb1* pathway. Taken together, our analyses indicate that *tb1* and *gt1* regulate complex changes in production and downstream signaling of multiple phytohormones, uncovering roles for both ABA and JA in promoting bud dormancy.

### TB1 regulates sugar levels and energy balance

**Fig. 4:**
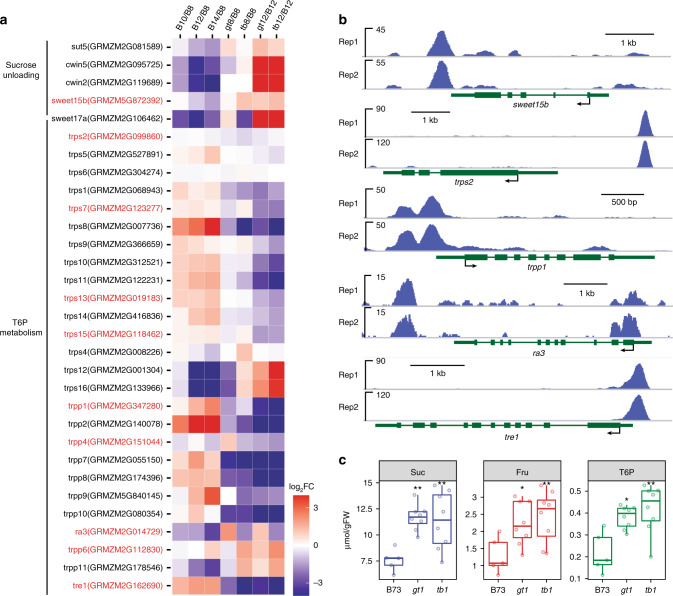
Bud dormancy is associated with *tb1*-*gt1-*mediated regulation of sugar signaling and energy homeostasis. **a** Genes involved in sucrose unloading and T6P metabolism were differentially expressed in tiller buds across B73 developmental series, as well as in *tb1* and *gt1* (bud stages and genotypes labeled as described in Fig. 3). Gene names highlighted in red were also bound by TB1 by ChIP-seq. FC fold change. Gradient color scale indicates the log value of expression fold change (log_2_FC). **b** TB1 ChIP-seq-binding peaks near several differentially expressed sugar signaling genes in **a**. rep1 and rep2 represent two biological replicates of the TB1 ChIP-seq assay. **c** Quantification of sucrose (Suc), fructose (Fru), and trehalose-6-phosphate (T6P) levels in tiller buds of B73, *gt1*, and *tb1* at 12 DAP. Plots show means ± SE calculated from at least five biological replicates. *p < 0.05, **p < 0.01; two-tailed Student’s *t*-test

A growing body of evidence indicates that metabolism of sugars and their associated intermediates are not only necessary for basic energy production, but also play crucial signaling roles during development^38^. This includes a likely role for sucrose in the breaking of bud dormancy following decapitation of the main shoot. When sucrose-induced bud reactivation was achieved in pea, it also resulted in down-regulation of its *tb1* ortholog^9^. Dormancy breaking is also associated with increased levels of trehalose 6-phosphate (T6P)^39^, highlighting an important signaling role for both sucrose and T6P in allowing bud growth. T6P is an intermediate in trehalose metabolism that acts as a signal of sucrose availability^40^. We were curious if *tb1*, in addition to responding to sugar signals, could directly modulate sugar signaling in the bud. Several genes known to promote sucrose unloading from phloem were up-regulated in *tb1* and *gt1*, including the *SWEET* hexose transporters, *SUCROSE TRANSPORTERs* (*SUT*) and *CELL WALL INVERTASE* (*CWIN*) (Fig. 4a). Of these, one *SWEET* transporter gene (*sweet15b*) was among the putative TB1-binding targets (Fig. 4b). Similarly, multiple TB1-bound targets associated with trehalose metabolism were differentially regulated in *tb1* and *gt1* buds, including *TREHALOSE-6-PHOSPHATE SYNTHETASE2* (*trps2*), *TREHALOSE-6-PHOSPHATE PHOSPHATASE1* (*trpp1*), *ramosa3* (*ra3*, a member of the *trpp* gene family), and *TREHALASE1* (*tre1*) (Fig. 4a and b). To confirm that these transcriptional changes had an effect on the accumulation of sugar molecules, we measured a variety of different metabolites involved in sugar and starch metabolism from dormant B73 and growing *tb1* and *gt1* buds. Consistent with the observed transcriptional changes, sucrose, fructose, and related intermediate metabolite levels were elevated in *tb1* and *gt1* buds, as was T6P (Fig. 4c and Supplementary Figs. 7–9). These results not only provide strong evidence that *tb1* and *gt1* negatively regulate sugar levels, but they may also down-regulate sugar signaling in wildtype through T6P, possibly to promote bud dormancy.

### TB1 integrates multiple domestication loci

**Fig. 5:**
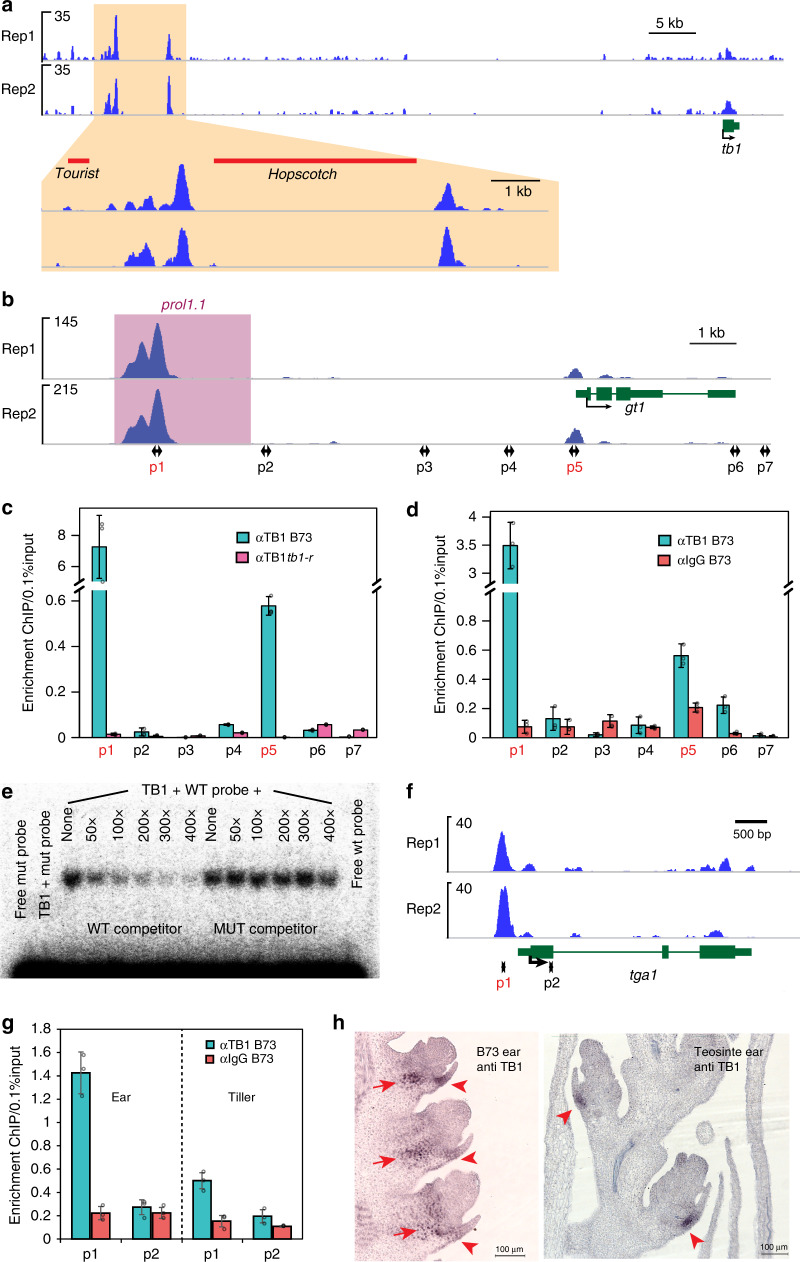
TB1 putatively targets several domestication loci. **a** TB1 binds to a cis-regulatory element region upstream of its own promoter. Two binding peaks localize to both sides of the *Hopscotch* retrotransposon responsible for domestication. **b** TB1 binds to the prolificacy locus (*prol1.1*, purple shade) in the *gt1* promoter. **c** and **d** ChIP qPCR using tiller buds **c** and ear tissue **d** to validate TB1 binding to the *prol1.1* QTL in both tissues. p1–p7 in **b** indicate the positions of the primers used for the ChIP qPCR. p1 and p5 primers in red indicate the primers showing significant enrichment in wild type. Values are means ± SD of three biological replicates. **e** Competition gel shift between TB1 protein with wild type versus mutated binding sites probes of the *prol1.1* sequence. **f** TB1 binds to the promoter of *tga1*. **g** Validation of TB1 binding to the *tga1* promoter using ChIP qPCR on both tiller bud and ear tissue. p1 and p2 in **f** indicate the positions of primers used for the ChIP qPCR. Values are means ± SD of three biological replicates. **h** TB1 immunolocalization on ear tissue of maize (left) compared to teosinte (right), showing TB1 accumulation in the developing glumes (arrowheads) is conserved between maize (left) and teosinte (right). However, stronger ectopic TB1 expression was observed (arrows) in maize. Bar = 100 microns

A major domestication QTL that drove selection at *tb1* was identified ~58 kb upstream of its own promoter where the insertion of a *HOPSCOTCH* retrotransposon caused overexpression in maize compared to teosinte^41^. Surprisingly, within this same domestication QTL, we observed two TB1-binding peaks flanking the *HOPSCOTCH* retrotransposon (Fig. 5a) at ~64 and ~57 kb, respectively, relative to the *tb1* coding region. A comparison of the sequences of both binding peaks in maize versus teosinte showed a very high degree of conservation (Supplementary Data 17), indicating that TB1 should be able to bind to this same region in teosinte. In light of the fact that most of the *tb1* DEGs are downregulated during early bud growth, we assume that *tb1* mainly functions as an activator, and thus positively auto-regulates its own expression. This is in line with the ectopic expression of TB1 observed in axillary branches in maize compared to teosinte^16^.

As predicted by a previous genetic analysis^17^, we found that TB1 putatively targets the domestication gene *gt1*, since the strongest, highest confidence TB1 ChIP peaks are found in the promoter of *gt1* (Fig. 5b). This is consistent with the role of *gt1* in suppressing tiller bud growth, an activity that overlaps with *tb1* function. Furthermore, *gt1* has been shown to have an additional branching function important for domestication. Teosinte normally produces primary lateral branches that terminate as male inflorescences, upon which multiple feminized secondary, and other higher order inflorescence branches are formed in the axils of leaves. In maize, however, these entire primary branches are replaced by single feminized unbranched ears, and the multiple feminized secondary inflorescences are suppressed, remaining as small dormant buds in the axils of husk leaves. The growth of these suppressed secondary branches in husk leaves has been described as prolificacy^19^, and suppression of this trait was critical during domestication. A recent study identified a major domestication QTL controlling prolificacy (*prol1.1)* within a region upstream of the promoter of *gt1*^19^. Interestingly, the highest confidence TB1 peak is located within the *gt1* promoter and maps to the exact location of the *prol1.1* (Fig. 5b)^19^. In addition, a second TB1-binding peak was found much closer to the protein coding region start site, though this peak is considerably smaller (Fig. 5b).

To validate these binding sites, ChIP-qPCR was performed on tiller bud chromatin from both wild type and *tb1* mutants. A series of primers were designed along the *gt1* genic region, and only those underlying both peaks showed significant enrichment (Fig. 5c). Furthermore, similar enrichment was also found in subsequent ChIP-qPCR using ear tissue (Fig. 5d), suggesting that the regulatory relationship between TB1 and *gt1* is conserved between tiller buds and ears. Since a GGNCCC motif was identified at the exact summit of the prolificacy peak, we used electrophoretic mobility shift assay (EMSA) to test if this motif is necessary for TB1 binding. As expected, probes carrying GGNCCC bound to TB1 in vitro, while those with mutated binding sites did not (Fig. 5e). Both RNA-seq and RT-qPCR revealed that *gt1* expression is decreased in the *tb1* mutant, indicating that *gt1* may be activated by TB1 binding as predicted (Supplementary Table 1, Supplementary Fig. 10A). The *tb1/gt1* double mutant showed the same level of tillering as the *tb1* mutant, confirming the epistatic relationship between *tb1* and *gt1* (Supplementary Fig. 10B–D).

We identified another domestication gene, *teosinte glume architecture 1* (*tga1*), as a putative TB1 target, consistent with a recent report^23^. *tga1* is responsible for the reduced, soft glumes found in modern maize compared to teosinte^42^. Despite the fact that *tga1* has a floral function and is expressed in floral tissue, a large TB1 ChIP peak was found in the promoter of *tga1* (Fig. 5f) using tiller bud tissue. This binding site was then confirmed by ChIP-qPCR using ChIP DNA from both tiller buds and ears, respectively (Fig. 5g), although stronger binding was observed in ears compared to tillers. Since *tb1* loss of function mutants do not make ears under our growth conditions, it is unclear whether the mutant displays any *tga1*-like ear phenotype. However, immunolocalization showed TB1 accumulation in wild type ear glumes (Fig. 5h) overlapping with the expression pattern of *tga1*^43^. A similar expression pattern was observed in teosinte ears, although in a smaller expression domain compared to maize (Fig. 5h). This is consistent with the increased TB1 protein levels and transcript observed in maize ears compared to teosinte^16^. Since the *tb1*, *gt1*, and *tga1* maize alleles are dominant to the teosinte alleles^23^, their putative positive direct regulation by the *Tb1* gain of function allele may be critical for their increased activity.

## Discussion

**Fig. 6:**
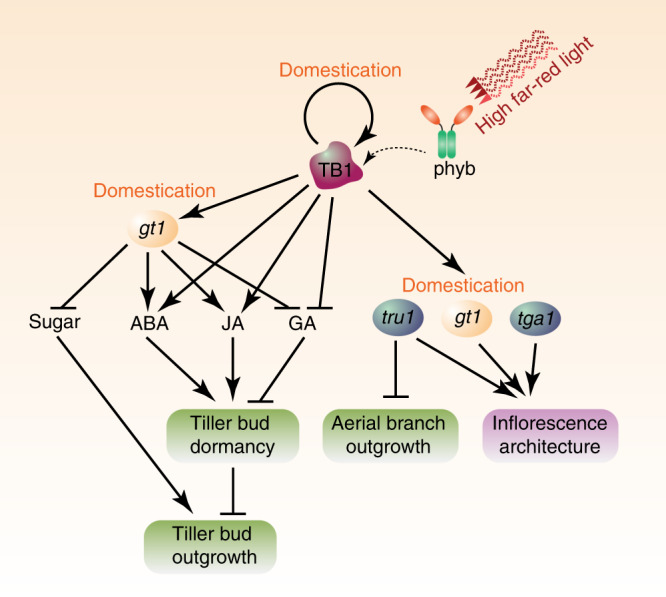
A model of the tiller regulatory network mediated by TB1. A high ratio of far-red light activates *tb1* expression. TB1 may reduce sugar levels by activating genes for downstream catabolism and signaling. TB1 also functions as a master regulator of phytohormones by positively regulating ABA and JA while negatively regulating GA. Integration of sugar and phytohormone dynamics may maintain dormancy of tiller buds and suppress their outgrowth. TB1 is predicted to directly modulate other domestication genes including *gt1*, *tru1*, and *tga1* that drove the dramatic morphological change from teosinte, positioning TB1 as a master regulator of the domestication hierarchy

Regulation of branching is critical for plants to adapt to diverse and changing environments. Here we describe the complex downstream genomic and transcriptomic responses, as well as classical plant hormone and sugar signaling, to the known activities of the maize branching regulators *tb1* and *gt1*. Direct measurements of phytohormones and sugar metabolites are consistent with genomic-binding targets and associated transcriptional changes in mutant tissues. Together, these responses reveal a crucial molecular switch that distinguishes growing from dormant lateral buds, and aspects of this regulatory network are functionally conserved in diverse plant lineages^24^. While both *tb1* and *gt1* were important targets of artificial selection during domestication, *tb1*, in particular, appears to be positioned at the top of a regulatory hierarchy that regulates a diverse set of maize domestication loci (Fig. 6).

We observed a more than two-fold reduction in the levels of the dormancy hormone, ABA in *tb1* and *gt1* axillary buds compared to wildtype (Fig. 3c). This reduction is consistent with the function of TB1 orthologs in eudicots, including the *Arabidopsis BRANCHED1* (*BRC1*) gene that was already implicated in bud dormancy and branch regulation^13,14^. Transcription profiling revealed that genes in the ABA pathway were significantly reduced in *brc1* mutants compared to wild type^44^. An analysis of *BRC1* targets^21^ showed that three closely related *gt1* co-orthologs (*HB40*, *HB53*, and *HB21*) were directly up-regulated by *BRC1*, and that all four transcription factors promoted expression of the ABA biosynthesis gene *NCED3*, leading to ABA accumulation and bud dormancy. Overall, this regulatory module appears to be largely conserved in maize, where *gt1* is a putative direct target of TB1 and both genes promote ABA biosynthesis and accumulation. In particular, we noted that *viviparous14* (*vp14*), a *NCED3* homolog^45^ that functions in ABA biosynthesis in maize^46^, is a putative TB1 target. Despite the large phylogenetic distance between monocots and eudicots, *tb1/gt1* induction of ABA to establish lateral bud dormancy appears to be a highly conserved module. ABA has a well-documented role in establishing seed dormancy in diverse seed plants including gymnosperms^47^, and it is possible that this conserved dormancy function of ABA was co-opted by *tb1* and *gt1* to regulate dormancy in the context of lateral buds in angiosperms.

In addition to ABA, we found that *tb1/gt1*-mediated bud arrest in wildtype is highly correlated with increased JA levels, since its levels are decreased more than 10-fold in buds of both *tb1* and *gt1* mutants. Several JA biosynthetic genes, including the classical maize sex determination gene *ts1*, and *OPR8* are differentially expressed in *tb1* and *gt1*, and putatively bound by TB1. In maize male tassel florets, carpel growth is aborted through the tissue-specific action of the *ts1* gene that encodes a lipoxygenase in the JA biosynthetic pathway^35^. Although JAs have not previously been implicated in the repression of tillering in maize, they have been shown to be involved in repression of reproductive axillary branch growth. Loss of function mutations in the JA biosynthetic genes *OPR7* and *OPR8*, the latter of which is a putative TB1 direct target, increased the number of nodes with lateral branches, as well as ear shank internode elongation^34^. This suggests a function for JAs in suppressing growth in axillary positions, and is consistent with the known effects of JA on suppression of mitosis^48^. These observations, however, contrast with a recent report in sorghum, where JA correlates with tiller bud growth rather than dormancy^33^, underscoring the need for more work on the role of JA in bud dormancy. Interestingly, another known target of TB1, *tru1*, also functions to suppress axillary branch elongation, as well as sex determination of the inflorescence, although it is not yet known whether this occurs through a JA-related mechanism.

Control of apical dominance and repression of bud growth is clearly an auxin-mediated effect, and yet multiple lines of evidence indicate that auxin does not act locally to maintain bud dormancy^3^. In support of this, we could not detect any difference in auxin levels between dormant wildtype and actively growing *tb1* and *gt1* buds. These data are consistent with a report in *Arabidopsis* that high FR:R and ABA suppress bud growth in a largely auxin-independent manner^49^. A major advance in the understanding of axillary bud suppression came from characterizing the sugar status of the bud and its source sink relationship with the nearby stem^50^. This alternative theory posits that instead of transported auxin, it is the availability of sucrose transported via the stem to sink tissue that is the primary determinant for apical dominance of bud growth^9^. Thus, removal of the terminal sink by decapitating the shoot apex and releasing the buds from apical dominance will allow sucrose to enter the dormant bud and activate growth. In line with this, high sucrose levels rapidly inhibit expression of the *tb1* ortholog *BRC1*, resulting in bud growth in pea plants^9^. We show, however, that *tb1* and *gt1* may also act upstream of sucrose, since they influence energy balance within repressed buds by putatively targeting sucrose transporters, sucrose catabolic genes, as well as sugar signaling via the T6P pathway. The increased expression of the putative TB1 target *sweet15b*, a sucrose transporter, could in part mediate the significant increase in sucrose levels in non-dormant *tb1* and *gt1* buds (Fig. 4a–c), as well as the other hexoses and tricarboxylic acid cycle intermediates (Supplementary Fig. 7). Furthermore, the high sucrose levels of *tb1* and *gt1* buds were associated with high T6P, whose metabolism and biosynthesis are also potentially targeted by TB1. In grasses, T6P levels regulate inflorescence branching as mutants of the T6P phosphatase *ramosa3*, another potential TB1 target, have increased inflorescence branching^51^, although it is not clear whether this gene may also be involved in regulation of transcription^52^. High T6P levels may be an indicator of high-energy status and sucrose availability^38^ both of which may be necessary for bud growth. According to the nexus model for T6P function^53^, high sucrose levels will be reflected by high T6P and thus promote growth. Conversely, plants with low levels of T6P have suppressed bud growth^54,55^, consistent with low levels of T6P seen in dormant B73 buds (Fig. 4C). Taken together, these data indicate that *gt1* and *tb1* are required in wildtype to maintain low sugar and T6P levels and thus are crucial regulators of carbohydrate levels and other sugar signals required for bud suppression.

A reduction in axillary branching is a key step towards designing agronomically useful crop plants^56^. Maize displays reduced axillary branching compared to its wild ancestor teosinte, but also morphological divergence in several other key domestication traits. These include a reduction and softening of the hard glumes and cupule, allowing easy access to the kernels, as well as a reduction in the number of female inflorescences produced per lateral branch, leading to less competition for resources per branch. Interestingly, *tb1* appears to be a common regulator of all of these diverse domestication phenotypes.

Previous work showed that the *tb1* domestication QTL has epistatic effects on several other domestication traits^57–59^. The TB1 ChIP seq data provides a simple explanation for this, since they show that the TB1 protein putatively binds to multiple domestication genes, including its own promoter. We assume that the TB1 autoregulation has a positive effect on its expression, since TB1 protein and transcript display ectopic overexpression in maize compared to teosinte (Fig. 5h)^12,16^. It is possible that this overexpression also has a similar positive effect on several known domestication loci, many of which also are gain of function alleles compared to teosinte. For example, many of the epistatic effects of *tb1* on lateral branch length and sex determination can be explained by its effect on binding to the *tru1* gene. The ectopic overexpression of *tb1* in domesticated maize ear shanks led to a similar expression pattern of *tru1*, causing reduced branch outgrowth and female sex identity in maize compared to teosinte^16^. Similarly, the previously described *tga1* maize domestication allele responsible for a reduction in glume hardness was shown to be dominant compared to teosinte, and associated with a single amino acid change at the N-terminal end of the protein^60^. *tga1* was recently shown to be a direct target of TB1 via binding to a GGNCCC motif upstream of the transcription start^23^, a result confirmed by our TB1 ChIP seq. We also show TB1 protein is localized to early glume primordia (Fig. 5h), consistent with the protein localization of TGA1 protein^43^, making their regulatory relationship feasible. Finally, previous work showed that the *gt1* domestication gene is genetically downstream of *tb1* in maize^17^, an observation consistent with TB1 ChIP where *gt1* was identified as the top enriched peak. This peak, located 7 kb upstream of the *gt1* promoter, corresponds to the location of another domestication QTL, *prol1.1*, that controls ear prolificacy^19^. Since this QTL is dominant to the teosinte allele^19^, it is possible that overexpression of the domesticated *Tb1* allele may have also led to overexpression of *gt1* via TB1 binding on *prol1.1*. Thus, all four maize alleles of cloned domestication/improvement loci, *tb1*, *gt1*, *tga1*, and *tru1*, are dominant compared to teosinte, and all are putatively bound by the domesticated TB1 protein. The predicted TB1 binding sites within these genes are tightly conserved in both maize and teosinte (Supplementary Data 17), suggesting that the modulation of TB1 activity on these genes is important for domestication as opposed to binding ability. Thus, it is possible that gain of function of the domesticated *Tb1* allele also led to gain of function of these target genes, although this will have to be confirmed in the case of *tga1* whose dominance appears to be dependent on an amino acid change^61^. Alternatively, it is possible that the initial selection for the dominant *Tb1* allele facilitated subsequent selection for the *tga1* and *gt1* domestication alleles. These possibilities may only be distinguished through a complete understanding of the timing of appearance of the *Tb1* allele compared to these other domestication target genes.

Even though *tb1* is genetically upstream of *gt1* in terms of regulating axillary branch outgrowth in both maize and Arabidopsis, this epistatic relationship does not appear to be fixed. In maize, both *tb1* and *gt1* function as repressors of tillering, but the mutants have phenotypes that are distinct, indicating that their respective pathways have diverged. For example, *gt1* is expressed in and required for repression of carpel growth in male florets^17^, while *tb1* is not. This suggests that both *tb1* and *gt1* can inhibit growth independently in different tissues. In light of this, it is intriguing that alleles of *tb1* and *gt1* have been selected for their effects on tissues other than vegetative axillary buds in multiple domesticated grasses. In barley, the switch from two-rowed to six-rowed inflorescences involved selection for mutations in the *tb1* ortholog *int-c*, as well as the *gt1* homolog *VrsI*^15,62^. Similarly, mutations in the *TB1* homolog of wheat led to an increase in spikelet branching in the inflorescence of several modern cultivars^63^, indicating that the gene normally represses floral branching in this species. Outside of the grasses, evolution of a novel regulatory mechanism that targets a *gt1* ortholog was responsible for inhibition of stamen growth and dioecy in persimmon^64,65^. Thus the utilization of *tb1* and *gt1* mediated growth suppression evolved repeatedly to reduce lateral organ number in diverse species and tissues. An intriguing unanswered question is how much of the *tb1/gt1* downstream network is conserved across species and tissues, and what if any are the consequences of integrating the distinct *tb1* and *gt1* networks.

The fact that homologs of *tb1* and *gt1* have consistently been selected to negatively regulate growth and expansion in various tissues indicate that they may comprise “hotspots of phenotypic variation”^66^. This view holds that developmental constraints, typically those structured by the morphogenic developmental networks, lead to the repeated selection of certain loci during evolution. Such loci represent genes that produce natural variants with large phenotypic effects and minimal pleiotropy. Master regulator genes that integrate diverse inputs to control multiple downstream effectors are predicted to be common genetic hotspots, assuming that regulatory (e.g. *cis*-element) variants can be produced that limit negative pleiotropy^67^. Both *tb1* and *gt1* conform to this expectation, being integrators of multiple inputs/outputs, and having complex promoters which were repeatedly modified and selected^19,20^. These genes have proven to be an effective module that can be re-employed to sculpt plant organ growth, with the potential for engineering agronomically beneficial growth in other contexts for which natural variation does not currently exist.

## Methods

### Plant materials and growth conditions

Both *tb1* mutant (*tb1-ref*) and *gt1* mutant (*gt1-1*) were introgressed in B73 at least five times. Plants used for RNA-seq assays were grown in controlled growth chambers at 24 °C, in 16-h light/8-h dark period. Since the *tb1-ref* allele cannot be propagated as homozygotes, a population that segregates *tb1-ref* homozygotes and *tb1-ref* heterozygotes in a ratio of 1:1 was used, and *tb1-ref* homozygotes in this population were identified by genomic PCR with *tb1*-specific primers (Supplementary Table 3).

To monitor early axillary tiller development, tiller buds in B73, *tb1* and *gt1* at 6, 8, 10, 12, 14, and 16 DAP were dissected from the first, second, and third leaf axil under a stereomicroscope, and the length of tiller buds measuring from the tip to the bottom of tiller buds was recorded.

### RNA-seq

Tiller buds from B73 at 8, 10, 12, and 14 DAP and from *tb1* and *gt1* at 8 and 12 DAP were dissected from the first leaf axil under stereomicroscope and immediately frozen in liquid nitrogen. All samplings were performed within a 2 h window in the afternoon to control for circadian effects. Depending on the size of tiller buds at a given developmental stage, 50–144 tiller buds were pooled per biological replicate; three to five biological replicates were collected per sample. Total RNA in tiller buds was isolated using a QIAGEN miRNeasy Micro kit (cat. no. 217084). RNA-seq libraries were prepared using a KAPA stranded mRNA kit (cat. no. KK8421) according to the manufacturer’s instruction (KAPABIOSYSTEMS, https://www.kapabiosystems.com). The average library insert size is ~500 bp. Libraries were quantified on an Agilent bioanalyzer (Agilent) and sequenced on an Illumina Hi-seq 2500 at Brigham Young University. Sequenced reads were 125 bp long with paired-ends (PEs).

### Differential expression analysis

In total, 27 RNA-seq libraries were sequenced and a total of ~1 billion PE raw reads were obtained with an average of 35 million reads per library. The overall quality of the sequencing data was assessed using FastQC^68^ and the raw reads were filtered using Trimmomatic v.0.36^69^ to trim and remove low-quality reads and adapter sequences. The filtered reads were mapped to maize B73 reference genome version 3, release 31 (AGPv3.31) using STAR aligner v.2.6.0a^70^ with default parameter settings. The total mapped reads and uniquely mapped reads are summarized in Supplementary Table 2. A read count matrix including all samples was generated by aggregating the raw counts of the mapped reads for a given gene in each sample using featureCounts^71^ with reference to 39,479 maize gene models in AGPv3.31. The read count matrix was subjected to differential gene expression analysis using the Bioconductor R package edgeR v.3.22.5^72^. EdgeR uses a generalized linear model (GLM) to identify differential enrichment by fitting the genomic count data to a negative binomial distribution. The GLM is implemented in glmQLfit and glmQLFTest functions in edgeR to identify DEGs between group comparison, and applied FDR and logFC cutoffs simultaneously to narrow down the genes that are biologically meaningful and differentially expressed. Briefly, genes with ubiquitously low expression were removed from the read count matrix in order to improve differentially expressed gene detection sensitivity and only the genes that had a count-per-million (CPM) value >1 in at least three libraries were retained. This resulted in a filtered read count matrix containing 23,343 expressed genes in our tiller bud samples (Supplementary Data 1). The filtered read count matrix was normalized for compositional bias between libraries using trimmed means of M values (TMM) method and then used to detect genes with differential expression between pairwise samples. Genes with an adjusted *p*-value (*q*-value) ≤0.05 and an absolute value of log2-fold change (FC) ≥ 1 were considered differentially expressed.

### Gene co-expression cluster analysis

The 6998 genes that exhibited differential expression both across B73 developmental series and between B73 and the two mutants (Supplementary Data 5) were subjected to co-expression cluster analysis using the Bioconductor R package coseq v1.5.2^25^. First, RPKM values (Reads Per Kilobase of transcript, per Million mapped reads) were extracted to form a gene expression matrix (Supplementary Data 18). The gene expression matrix was then used as an input in coseqR. Log CLR-transformation and TMM normalization were applied to the gene expression matrix to normalize the expression of genes and the *K*-means algorithm was chosen to detect the co-expressed clusters across all samples. The *K*-mean algorithm was repeated 15 times in order to determine the optimal number of clusters. The resulting number of clusters in each run was recorded, and the most parsimonious cluster partition was selected using adjusted random index (compareARI function in coseq). Finally, genes that were assigned to clusters with maximum conditional probability of ≥0.8 were retained for cluster visualization and gene ontology (GO)-enrichment analysis per each cluster.

### GO-enrichment analysis

Statistically enriched (adjusted *p*-value ≤ 0.05) GO terms on genes that are differentially expressed between pairwise samples or on genes assigned to each co-expression cluster were identified using singular enrichment analysis (SEA) in AgriGO v2.0^73^. SEA analysis was based on the suggested background of maize B73 reference genome AGPv3. Statistical testing was performed based on the default options of Fisher test and FDR adjustment method. The most statistically enriched GO terms were visualized in ggplot2^74^.

### ChIP

Maize B73 and *tb1-r* plants were grown in the experimental field of the Plant Gene Expression Center, UC Berkeley. Three to five tiller buds per plant were carefully dissected from 2.5 to 3 weeks old B73 and *tb1-r* homolog mutants, after genotyping the *tb1-r* families. Tissue was cross-linked for 10 min in 1% formaldehyde solution under vacuum, and quenched by adding glycine to a final concentration of 0.1 M. Cross-linked materials were frozen in liquid nitrogen, ground and suspended in chromatin extraction buffer 1 (10 mM Tris–HCl (pH 8.0), 0.4 M sucrose, 10 mM MgCl_2_, 1 mM phenylmethylsulfonyl fluoride (PMSF), 5 mM β-mercaptoethanol, and 1× Complete Protease Inhibitor Cocktail), and filtered through two layers of mesh (50-μm pore size). The nuclei pellets were washed using extraction buffer 2 (10 mM Tris–HCl (pH 8.0), 0.25 M sucrose, 10 mM MgCl_2_, 1% Triton X-100, 1 mM PMSF, 5 mM β-mercaptoethanol, and 1× Complete Protease Inhibitor Cocktail) and extraction buffer 3 (10 mM Tris–HCl (pH 8.0), 1.7 M sucrose, 2 mM MgCl_2_, 0.15% Triton X-100, 1 mM PMSF, 5 mM β-mercaptoethanol, and 1× Complete Protease Inhibitor Cocktail). Isolated chromatin complex were sheared in 300 μL of nuclei lysis buffer (50 mM Tris–HCl (pH 8.0), 10 mM EDTA, 1% (w/v) SDS, 1 mM PMSF, and 1× Complete Protease Inhibitor Cocktail) until the average genomic DNA fragment size of ~200–500 bp using a sonicator (Bioruptor). 150 μl fragmented chromatin was diluted with 1350 μl of ChIP dilution buffer (50 mM Tris–HCl (pH 8.0), 167 mM NaCl, 1.1% Triton X-100, 0.11% sodium deoxycholate, and 1× Complete Protease Inhibitor Cocktail). Dynabeads Protein A magnetic beads (Invitrogen) were blocked in blocking buffer (200 μg Glycogen, 200 μg BSA and 200 μg Yeast tRNA in 1 mL ChIP dilution buffer). Isolated chromatin were precleared by incubating with 40 μL blocked Dynabeads for 2 h at 4 °C. Anti-TB1 antibody was produced and purified by our group previously^16^. Normal guinea pig IgG (Santa Cruz Biotechnology, sc-2711) was used as a negative control. ~2 μg of TB1 antibody or IgG was used in the immunoprecipitation reactions at 4 °C overnight. The bound chromatin complex was captured by 40 μl blocked Dynabeads, and washed successively with low-salt wash buffer (50 mM Tris–HCl, pH 8.0, 150 mM NaCl, 1 mM EDTA, 0.1% SDS, 0.1% sodium deoxycholate, 1% Triton X-100, and 1× Complete Protease Inhibitor Cocktail), high-salt wash buffer (50 mM Tris–HCl, pH 8.0, 500 mM NaCl, 1 mM EDTA, 0.1% SDS, 0.1% sodium deoxycholate, 1% Triton X-100, and 1× Complete Protease Inhibitor Cocktail), lithium chloride buffer (250 mM LiCl, 1% Nonidet P-40, 1% sodium deoxycholate, 1 mM EDTA, and 10 mM Tris–HCl, pH 8.0), and two times with Tris–EDTA buffer. The beads pellets were incubated at 65 °C overnight in 200 μL of elution buffer (10 mM Tris–HCl, pH 8.0, 300 mM NaCl, 5 mM EDTA, 0.5% SDS, 20 mg of Proteinase K and 3 μl RNaseA). ChIP DNA was isolated by phenol–chloroform extraction and isopropanol precipitation with 40 μg of glycogen (Roche) and 240 mM sodium acetate. Approximately 2 g of tiller buds were used for each ChIP experiment, and the yield DNA from five experiments was pooled as one replicate for ChIP-seq library construction. The NEXTflex ChIP-Seq Kit (Bioo Scientific, NOVA-5143-01) was used for library construction according to the manufacturer’s protocol. Fourteen PCR cycles were performed for library amplification.

### ChIP-seq

The ChIP-seq DNA libraries were quality checked by bioanalyzer and quantified by Qubit and qPCR, and sequenced using the Illumina Hiseq 4000 platform, generating 50 bp single end reads. The reads were aligned to the maize genome (Zea_mays.AGPv3.22) allowing one mismatch (-n 1) using Bowtie2^75^. Only uniquely mapped reads with MAQ20 (map quality > 20) by SAMtools were used for the following peak calling. MACS2 software (version 2.1.0) (https://github.com/taoliu/MACS) was used for peak calling with the genome size of –g 2.1e + 9 and with default settings of cut off *q* value (<0.05). Significant peaks from two biological replicates relative to the IgG control samples were identified as reproducible peaks if their summits were positioned within 300 bp of each other. By using the intersect intervals function of BEDTools (version 2.26.0)^76^, we assigned the putative TB1 target genes if reproducible peaks localize in the range from 10 kb upstream to 5 kb downstream of the gene. The bigwig files of MACS2 output were visualized using the Integrated Genomics Viewer (v.2.3.90)^77^. One hundred base pairs around the reproducible peak summits (upstream 50 bp and downstream 50 bp) were extracted and performed motif enrichment analysis using the MEME program (version 4.11.2)^78^. The most enriched motif identified were kept for further candidate as the major TB1-binding motif.

### ChIP-qPCR

The same type of tiller bud tissue and <5 mm ear tissue was harvested for the ChIP-qPCR experiment to validate the putative TB1-binding targets. Three biological replicates of immunoprecipitated DNA in ChIP was applied for each qPCR using respective primer pairs listed in Supplementary Table 3 with Fast Evagreen qPCR mix. Levels were calculated using the ΔCt (threshold cycle) method. Enrichment levels were normalized to the 1% input sample, and IgG served as a negative control.

### Gene expression level determined by qRT-PCR

Total RNA was isolated from tiller buds or ears harvested as described for ChIP. For qRT-PCR analysis, cDNA was synthesized from DNase I-treated total RNA using SuperScript III First-Strand Synthesis System (Invitrogen), and then diluted by 10-fold before using as template in 20 μl qPCR reaction mix. The standard deviation was calculated among three biological replicates for each sample. Maize Actin1 was used as the internal reference to normalize the expression data. The primers used for qPCR are listed in Supplementary Table 3.

### Immunolocalization

2 weeks old shoot or <1 cm ear tissue was embedded in paraplast plus (Sigma-P3683), and standard paraffin sections were dewaxed in Histoclear and rehydrated in ethanol–water gradient^16,79^. Then the slides were immersed in boiled 10 mM sodium citrate buffer, pH 6.0 for 3 min to retrieve epitope, and further blocked in Blocking Reagent (1x PBS, 2 mg/mL powder milk, and 0.1% Triton X-100). A 1:200 dilution of TB1 antibody was added to the slides and incubated overnight at 4 °C. After three washes in blocking solution, an anti-guinea pig alkaline phosphatase AP-conjugated secondary antibody was added (Invitrogen Cat # A18772) and incubated at room temperature for 1 h. After washing three times as above, the slides were immersed in TNM for 10 min before developing in Developing solution (20 μL of NBT-BCIP in 1 mL of 1x TNM buffer). As the signal is visible, immerse the slides in water to stop the reaction.

### EMSA

The 5′ truncation of TB1 including the TCP domain was cloned into the pDEST15 and expressed in *Escherichia coli*. Recombinant protein was purified using Glutathione Sepharose beads. Approximately 100  ng of recombinant protein was added to 1 pmol of double-stranded oligonucleotide probes labeled with gamma ATP using polynucleotide kinase and run on nondenaturing PAGE gels. For competition experiments, 50, 100, 200, 300, and 400 pmol, respectively, of unlabeled competitor oligo was added to the binding reactions.

### Phytohormone measurement in tiller buds

Limited by the amount of tissues (100 mg) needed for phytohormone profiling analysis, we only collected axillary buds from B73, *tb1*, and *gt1* at 12 DAP for hormone profiling. Three biological replicates per sample were used in the analysis. Tiller buds were dissected and frozen immediately in liquid nitrogen and stored at −80 °C. Frozen plant samples were extracted with 900 µL of ice-cold acetonitrile/methanol (1:1 v:v) while keeping samples cold on ice. We spike in 10 µl of a mixture of heavy isotope-labeled phytohormone standards at the beginning of the extraction (D, 13C, 15N) to serve as internal standards for quantitation and normalization of the samples to account for differing extraction efficiencies and day to day instrumental variations. Our standard assay is for IAA, JA, JA-Ile, OPDA, ABA. Two stainless steel 5 mm beads were added to each sample tube followed by brief mixing. Samples were placed in pre-cooled (−80 °C) TissueLyserII racks and homogenized for 2 min at 15 Hz. Samples were centrifuged at full speed for 5 min at 4 °C, then the supernatant was transferred to a new 2 mL tube. The samples were extracted with another 900 µL of extraction solvent, and then homogenized again for 2 min at 15 Hz. Two extractions were pooled, and extraction solvent was removed under reduced pressure with a speed-vac until completely dry. Samples were reconstituted in 30% MeOH (200 µL) and mixed thoroughly for 30 min at 4 °C. Samples were filtered through 0.8 µm PES spin-filters and 40 µL of clarified supernatant was transferred to HPLC vials. 2 µL of clarified supernatant were subjected to high-performance reverse-phase liquid chromatography–tandem mass spectrometry (HPLC–MS/MS) for detection and quantitation of phytohormones. Briefly, clarified samples were analyzed on an Eksigent ekspert™ microLC200 coupled to a Sciex 6500 QTrap^®^ (Framingham, MA) operated in multiple reaction monitoring (MRM) mode employing polarity-switching for simultaneous detection of positive and negative ions. The LC separation was achieved using a Waters (Milford, MA) Acquity UPLC^®^ BEH C18 1.0 × 100 mm, 1.7 µm column kept at 50 °C with a flow rate of 15 µL/min while the autosampler was set at 8 °C. The mobile phases were 0.1% acetic acid (mobile phase “A”) and 3:1 acetonitrile:methanol (mobile phase “B”) containing 0.1% acetic acid running a gradient of 20% “B” for 4 min ramping to 70% “B” at 7 min, increase to 95% “B” at 7.5 min, holding for 5.5 min, then re-equilibrate at initial conditions at 13.5 min for 10 min (total runtime is 23.5 min). Data analysis was completed using MultiQuant 3.0.2 (AB Sciex).

### Sucrose-related metabolite profiling analysis in tiller buds

The tiller buds from B73, *tb1* and *gt1* at 12 DAP were dissected from the first leaf axil under stereomicroscope and immediately frozen into liquid nitrogen for later use. All samplings were performed within a 2 h window in the afternoon to control for circadian effects. 15–25 mg of tiller buds were collected per biological replicate; each biological replicate is a pool of 20–115 tiller buds; five to eight biological replicates were collected per sample. Frozen tiller buds were ground to a fine powder under liquid nitrogen and were extracted with chloroform–methanol.T6P, other phosphorylated intermediates and organic acids were measured and quantified by liquid chromatography–tandem mass spectrometry (LC–MS/MS) and metabolites were quantified by comparison of the integrated MS-Q3 signal peak area with a calibration curve obtained using authentic standards^40^. Sucrose, glucose, fructose, and starch were measured enzymatically in the soluble and residual fractions of an ethanol/water extraction^80^.

### Reporting summary

Further information on research design is available in the Nature Research Reporting Summary linked to this article.

## Supplementary information

Supplementary Information

Description of Additional Supplementary Files

Supplementary Data 1

Supplementary Data 2

Supplementary Data 3

Supplementary Data 4

Supplementary Data 5

Supplementary Data 6

Supplementary Data 7

Supplementary Data 8

Supplementary Data 9

Supplementary Data 10

Supplementary Data 11

Supplementary Data 12

Supplementary Data 13

Supplementary Data 14

Supplementary Data 15

Supplementary Data 16

Supplementary Data 17

Supplementary Data 18

Reporting Summary

## Source data

Source Data

## Data Availability

All RNA-seq and ChIP-seq data generated in this study have been deposited in the National Center for Biotechnology Information Sequence Read Archive (accession code PRJNA517683). All other relevant data supporting the key findings of this study are available within the article and its Supplementary Information files or from the corresponding author upon reasonable request. The source data underlying Figs. 1a, 3c, 4c, 5c–e, g, Supplementary Fig. 1, Supplementary Fig. 6A, Supplementary Fig. 7, and Supplementary Fig. 110a, c, d are provided as a Source Data file.

## References

[1] Doebley, J., Stec, A., Wendel, J. & Edwards, M. Genetic and morphological analysis of a maize-teosinte F2 population: implications for the origin of maize. *Proc. Natl Acad. Sci. USA* **87**, 9888–9892 (1990). 11607138 10.1073/pnas.87.24.9888 PMC55279

[2] Gallavotti, A. The role of auxin in shaping shoot architecture. *J. Exp. Bot.* **64**, 2593–2608 (2013). 23709672 10.1093/jxb/ert141

[3] Domagalska, M. A. & Leyser, O. Signal integration in the control of shoot branching. *Nat. Rev. Mol. Cell Biol.* **12**, 211–221 (2011). 21427763 10.1038/nrm3088

[4] Dun, E. A., de Saint Germain, A., Rameau, C. & Beveridge, C. A. Antagonistic action of strigolactone and cytokinin in bud outgrowth control. *Plant Physiol.* **158**, 487–498 (2012). 22042819 10.1104/pp.111.186783 PMC3252097

[5] Ferguson, B. J. & Beveridge, C. A. Roles for auxin, cytokinin, and strigolactone in regulating shoot branching. *Plant Physiol.* **149**, 1929–1944 (2009). 19218361 10.1104/pp.109.135475 PMC2663762

[6] Gomez-Roldan, V. et al. Strigolactone inhibition of shoot branching. *Nature* **455**, 189–194 (2008). 18690209 10.1038/nature07271

[7] Sachs, T. & Thimann, K. V. The role of auxins and cytokinins in the release of buds from dominance. *Am. J. Bot.* **54**, 136–144 (1967).

[8] Booker, J., Chatfield, S. & Leyser, O. Auxin acts in xylem-associated or medullary cells to mediate apical dominance. *Plant Cell* **15**, 495–507 (2003). 12566587 10.1105/tpc.007542 PMC141216

[9] Mason, M. G., Ross, J. J., Babst, B. A., Wienclaw, B. N. & Beveridge, C. A. Sugar demand, not auxin, is the initial regulator of apical dominance. *Proc. Natl Acad. Sci. USA* **111**, 6092–6097 (2014). 24711430 10.1073/pnas.1322045111 PMC4000805

[10] Barbier, F. et al. Sucrose is an early modulator of the key hormonal mechanisms controlling bud outgrowth in *Rosa hybrida*. *J. Exp. Bot.* **66**, 2569–2582 (2015). 25873679 10.1093/jxb/erv047 PMC4986866

[11] Eveland, A. L. & Jackson, D. P. Sugars, signalling, and plant development. *J. Exp. Bot.* **63**, 3367–3377 (2012). 22140246 10.1093/jxb/err379

[12] Doebley, J., Stec, A. & Hubbard, L. The evolution of apical dominance in maize. *Nature* **386**, 485–488 (1997). 9087405 10.1038/386485a0

[13] Aguilar-Martínez, J. A., Poza-Carrión, C. & Cubas, P. Arabidopsis BRANCHED1 acts as an integrator of branching signals within axillary buds. *Plant Cell* **19**, 458–472 (2007). 17307924 10.1105/tpc.106.048934 PMC1867329

[14] Finlayson, S. A. Arabidopsis Teosinte Branched1-like 1 regulates axillary bud outgrowth and is homologous to monocot Teosinte Branched1. *Plant Cell Physiol.* **48**, 667–677 (2007). 17452340 10.1093/pcp/pcm044

[15] Ramsay, L. et al. INTERMEDIUM-C, a modifier of lateral spikelet fertility in barley, is an ortholog of the maize domestication gene TEOSINTE BRANCHED 1. *Nat. Genet.* **43**, 169–172 (2011). 21217754 10.1038/ng.745

[16] Dong, Z. et al. Ideal crop plant architecture is mediated by tassels replace upper ears1, a BTB/POZ ankyrin repeat gene directly targeted by TEOSINTE BRANCHED1. *Proc. Natl Acad. Sci. USA* **114**, E8656–E8664 (2017). 28973898 10.1073/pnas.1714960114 PMC5642732

[17] Whipple, C. J. et al. grassy tillers1 promotes apical dominance in maize and responds to shade signals in the grasses. *Proc. Natl Acad. Sci. USA* **108**, E506–E512 (2011). 21808030 10.1073/pnas.1102819108 PMC3158142

[18] Kebrom, T. H., Burson, B. L. & Finlayson, S. A. Phytochrome B represses Teosinte Branched1 expression and induces sorghum axillary bud outgrowth in response to light signals. *Plant Physiol.* **140**, 1109–1117 (2006). 16443694 10.1104/pp.105.074856 PMC1400571

[19] Wills, D. M. et al. From many, one: genetic control of prolificacy during maize domestication. *PLoS Genet.* **9**, e1003604 (2013). 23825971 10.1371/journal.pgen.1003604 PMC3694832

[20] Clark, R. M., Wagler, T. N., Quijada, P. & Doebley, J. A distant upstream enhancer at the maize domestication gene tb1 has pleiotropic effects on plant and inflorescent architecture. *Nat. Genet.* **38**, 594–597 (2006). 16642024 10.1038/ng1784

[21] González-Grandío, E. et al. Abscisic acid signaling is controlled by a BRANCHED1/HD-ZIP I cascade in Arabidopsis axillary buds. *Proc. Natl Acad. Sci. USA* **114**, E245–E254 (2017). 28028241 10.1073/pnas.1613199114 PMC5240681

[22] Yao, C. & Finlayson, S. A. Abscisic acid is a general negative regulator of Arabidopsis axillary bud growth. *Plant Physiol.* **169**, 611–626 (2015). 26149576 10.1104/pp.15.00682 PMC4577412

[23] Studer, A. J., Wang, H. & Doebley, J. F. Selection during maize domestication targeted a gene network controlling plant and inflorescence architecture. *Genetics* **207**, 755–765 (2017). 28754660 10.1534/genetics.117.300071 PMC5629337

[24] Dong, Z., Alexander, M. & Chuck, G. Understanding grass domestication through maize mutants. *Trends Genet.* **35**, 118–128 (2019). 30509788 10.1016/j.tig.2018.10.007

[25] Rau, A. & Maugis-Rabusseau, C. Transformation and model choice for RNA-seq co-expression analysis. *Brief. Bioinform.* **19**, 425–436 (2018). 28065917 10.1093/bib/bbw128

[26] Tsuda, K., Kurata, N., Ohyanagi, H. & Hake, S. Genome-wide study of KNOX regulatory network reveals brassinosteroid catabolic genes important for shoot meristem function in rice. *Plant Cell* **26**, 3488–3500 (2014). 25194027 10.1105/tpc.114.129122 PMC4213158

[27] Kosugi, S. & Ohashi, Y. DNA binding and dimerization specificity and potential targets for the TCP protein family. *Plant J.* **30**, 337–348 (2002). 12000681 10.1046/j.1365-313x.2002.01294.x

[28] Kang, J. et al. PDR-type ABC transporter mediates cellular uptake of the phytohormone abscisic acid. *Proc. Natl Acad. Sci. USA* **107**, 2355–2360 (2010). 20133880 10.1073/pnas.0909222107 PMC2836657

[29] Kuromori, T. et al. ABC transporter AtABCG25 is involved in abscisic acid transport and responses. *Proc. Natl Acad. Sci. USA* **107**, 2361–2366 (2010). 20133881 10.1073/pnas.0912516107 PMC2836683

[30] Brugière, N. et al. Overexpression of RING domain E3 ligase ZmXerico1 confers drought tolerance through regulation of ABA homeostasis. *Plant Physiol.* **175**, 1350–1369 (2017). 28899960 10.1104/pp.17.01072 PMC5664481

[31] Rubio-Moraga, A. et al. Apical dominance in saffron and the involvement of the branching enzymes CCD7 and CCD8 in the control of bud sprouting. *BMC Plant Biol.* **14**, 171 (2014). 24947472 10.1186/1471-2229-14-171 PMC4077219

[32] Luo, L. et al. Developmental analysis of the early steps in strigolactone‐mediated axillary bud dormancy in rice. *Plant J.* **97**, 1006–1021 (2019). 30740793 10.1111/tpj.14266 PMC6850044

[33] Liu, R. & Finlayson, S. A. Sorghum tiller bud growth is repressed by contact with the overlying leaf. *Plant Cell Environ.* **2**, 2120–2132 (2019). 10.1111/pce.13548 30875440

[34] Yan, Y. et al. Disruption of OPR7 and OPR8 reveals the versatile functions of jasmonic acid in maize development and defense. *Plant Cell* **24**, 1420–1436 (2012). 22523204 10.1105/tpc.111.094151 PMC3398555

[35] Acosta, I. F. et al. tasselseed1 is a lipoxygenase affecting jasmonic acid signaling in sex determination of maize. *Science* **323**, 262–265 (2009). 19131630 10.1126/science.1164645

[36] Gupta, R. & Chakrabarty, S. K. Gibberellic acid in plant: still a mystery unresolved. *Plant Signal. Behav.* **8**, e25504 (2013). 23857350 10.4161/psb.25504 PMC4002599

[37] Hedden, P. & Sponsel, V. A century of gibberellin research. *J. Plant Growth Regul.* **34**, 740–760 (2015). 26523085 10.1007/s00344-015-9546-1 PMC4622167

[38] Wingler, A. Transitioning to the next phase: the role of sugar signaling throughout the plant life cycle. *Plant Physiol.* **176**, 1075–1084 (2018). 28974627 10.1104/pp.17.01229 PMC5813577

[39] Fichtner, F. et al. Trehalose 6-phosphate is involved in triggering axillary bud outgrowth in garden pea (*Pisum sativum* L.). *Plant J.* **92**, 611–623 (2017). 28869799 10.1111/tpj.13705

[40] Figueroa, C. M. & Lunn, J. E. A tale of two sugars: trehalose 6-phosphate and sucrose. *Plant Physiol.* **172**, 7–27 (2016). 27482078 10.1104/pp.16.00417 PMC5074632

[41] Studer, A., Zhao, Q., Ross-Ibarra, J. & Doebley, J. Identification of a functional transposon insertion in the maize domestication gene tb1. *Nat. Genet.* **43**, 1160–1163 (2011). 21946354 10.1038/ng.942 PMC3686474

[42] Dorweiler, J., Stec, A., Kermicle, J. & Doebley, J. Teosinte glume architecture 1: a genetic locus controlling a key step in maize evolution. *Science* **262**, 233–235 (1993). 17841871 10.1126/science.262.5131.233

[43] Preston, J. C., Wang, H., Kursel, L., Doebley, J. & Kellogg, E. A. The role of teosinte glume architecture (tga1) in coordinated regulation and evolution of grass glumes and inflorescence axes. *New Phytol.* **193**, 204–215 (2012). 21954998 10.1111/j.1469-8137.2011.03908.x

[44] Gonzalez-Grandio, E., Poza-Carrion, C., Sorzano, C. O. S. & Cubas, P. BRANCHED1 promotes axillary bud dormancy in response to shade in Arabidopsis. *Plant Cell* **25**, 834–850 (2013). 23524661 10.1105/tpc.112.108480 PMC3634692

[45] Tan, B.-C. et al. Molecular characterization of the Arabidopsis 9-cis epoxycarotenoid dioxygenase gene family. *Plant J.* **35**, 44–56 (2003). 12834401 10.1046/j.1365-313x.2003.01786.x

[46] Xiong, L. & Zhu, J.-K. Regulation of abscisic acid biosynthesis. *Plant Physiol.* **133**, 29–36 (2003). 12970472 10.1104/pp.103.025395 PMC523868

[47] FeurtadoJ. A. & KermodeA. R. A merging of paths: abscisic acid and hormonal cross-talk in the control of seed dormancy maintenance and alleviation. *Annu. Plant Rev.* **27**, 176–223 (2007).

[48] Zhang, Y. & Turner, J. G. Wound-induced endogenous jasmonates stunt plant growth by inhibiting mitosis. *PLoS One* **3**, e3699 (2008). 19002244 10.1371/journal.pone.0003699 PMC2577035

[49] Holalu, S. V. & Finlayson, S. A. The ratio of red light to far red light alters Arabidopsis axillary bud growth and abscisic acid signalling before stem auxin changes. *J. Exp. Bot.* **68**, 943–952 (2017). 28062593 10.1093/jxb/erw479 PMC5444464

[50] Kebrom, T. H. A growing stem inhibits bud outgrowth—the overlooked theory of apical dominance. *Front. Plant Sci.* **8**, 1874 (2017). 29163599 10.3389/fpls.2017.01874 PMC5671643

[51] Satoh-Nagasawa, N., Nagasawa, N., Malcomber, S., Sakai, H. & Jackson, D. A trehalose metabolic enzyme controls inflorescence architecture in maize. *Nature* **441**, 227–230 (2006). 16688177 10.1038/nature04725

[52] Eveland, A. L. et al. Regulatory modules controlling maize inflorescence architecture. *Genome Res.* **24**, 431–443 (2014). 24307553 10.1101/gr.166397.113 PMC3941108

[53] Yadav, U. P. et al. The sucrose–trehalose 6-phosphate (Tre6P) nexus: specificity and mechanisms of sucrose signalling by Tre6P. *J. Exp. Bot.* **65**, 1051–1068 (2014). 24420566 10.1093/jxb/ert457 PMC3935566

[54] Schluepmann, H., Pellny, T., van Dijken, A., Smeekens, S. & Paul, M. Trehalose 6-phosphate is indispensable for carbohydrate utilization and growth in Arabidopsis thaliana. *Proc. Natl Acad. Sci. USA* **100**, 6849–6854 (2003). 12748379 10.1073/pnas.1132018100 PMC164535

[55] van Dijken, A. J. H., Schluepmann, H. & Smeekens, S. C. M. Arabidopsis trehalose-6-phosphate synthase 1 is essential for normal vegetative growth and transition to flowering. *Plant Physiol.* **135**, 969–977 (2004). 15181208 10.1104/pp.104.039743 PMC514131

[56] Harlan, J. R., de Wet, J. M. J. & Price, E. G. Comparative evolution of cereals. *Evolution* **27**, 311–325 (1973). 28564784 10.1111/j.1558-5646.1973.tb00676.x

[57] Doebley, J. & Stec, A. Inheritance of the morphological differences between maize and teosinte: comparison of results for two F2 populations. *Genetics* **134**, 559–570 (1993). 8325489 10.1093/genetics/134.2.559 PMC1205498

[58] Briggs, W. H., McMullen, M. D., Gaut, B. S. & Doebley, J. Linkage mapping of domestication loci in a large maize teosinte backcross resource. *Genetics* **177**, 1915–1928 (2007). 17947434 10.1534/genetics.107.076497 PMC2147989

[59] Studer, A. J. & Doebley, J. F. Evidence for a natural allelic series at the maize domestication locus teosinte branched1. *Genetics* **191**, 951–958 (2012). 22505628 10.1534/genetics.112.138479 PMC3389986

[60] Wang, H. et al. The origin of the naked grains of maize. *Nature* **436**, 714–719 (2005). 16079849 10.1038/nature03863 PMC1464477

[61] Wang, H., Studer, A. J., Zhao, Q., Meeley, R. & Doebley, J. F. Evidence that the origin of naked kernels during maize domestication was caused by a single amino acid substitution in tga1. *Genetics* **200**, 965–974 (2015). 25943393 10.1534/genetics.115.175752 PMC4512555

[62] Komatsuda, T. et al. Six-rowed barley originated from a mutation in a homeodomain-leucine zipper I-class homeobox gene. *Proc. Natl Acad. Sci. USA* **104**, 1424–1429 (2007). 17220272 10.1073/pnas.0608580104 PMC1783110

[63] Dixon, L. E. et al. TEOSINTE BRANCHED1 regulates inflorescence architecture and development in bread wheat (*Triticum aestivum*). *Plant Cell* **30**, 563–581 (2018). 29444813 10.1105/tpc.17.00961 PMC5894836

[64] Akagi, T., Henry, I. M., Kawai, T., Comai, L. & Tao, R. Epigenetic regulation of the sex determination gene MeGI in polyploid persimmon. *Plant Cell* **28**, 2905–2915 (2016). 27956470 10.1105/tpc.16.00532 PMC5240738

[65] Akagi, T., Henry, I. M., Tao, R. & Comai, L. A Y-chromosome-encoded small RNA acts as a sex determinant in persimmons. *Science* **346**, 646–650 (2014). 25359977 10.1126/science.1257225

[66] Martin, A. & Orgogozo, V. The Loci of repeated evolution: a catalog of genetic hotspots of phenotypic variation. *Evolution* **67**, 1235–1250 (2013). 23617905 10.1111/evo.12081

[67] Stern, D. L. & Orgogozo, V. The loci of evolution: how predictable is genetic evolution? *Evolution* **62**, 2155–2177 (2008). 18616572 10.1111/j.1558-5646.2008.00450.x PMC2613234

[68] Andrews, S. *FastQC: A Quality Control Tool for High Throughput Sequence Data*. http://www.bioinformatics.babraham.ac.uk/projects/fastqc (2010).

[69] Bolger, A. M., Lohse, M. & Usadel, B. Trimmomatic: a flexible trimmer for Illumina sequence data. *Bioinformatics* **30**, 2114–2120 (2014). 24695404 10.1093/bioinformatics/btu170 PMC4103590

[70] Dobin, A. & Gingeras, T. R. Mapping RNA-seq reads with STAR. *Curr. Protoc. Bioinforma.* **51**, 11.14.1–19 (2015). 10.1002/0471250953.bi1114s51 PMC4631051 26334920

[71] Liao, Y., Smyth, G. K. & Shi, W. featureCounts: an efficient general purpose program for assigning sequence reads to genomic features. *Bioinformatics* **30**, 923–930 (2014). 24227677 10.1093/bioinformatics/btt656

[72] Robinson, M. D., McCarthy, D. J. & Smyth, G. K. edgeR: a Bioconductor package for differential expression analysis of digital gene expression data. *Bioinformatics* **26**, 139–140 (2010). 19910308 10.1093/bioinformatics/btp616 PMC2796818

[73] Tian, T. et al. agriGO v2.0: a GO analysis toolkit for the agricultural community, 2017 update. *Nucleic Acids Res.* **45**, W122–W129 (2017). 28472432 10.1093/nar/gkx382 PMC5793732

[74] Wickham, H. *ggplot2: Elegant Graphics for Data Analysis* (Springer, New York, 2016).

[75] Langmead, B. & Salzberg, S. L. Fast gapped-read alignment with Bowtie 2. *Nat. Methods* **9**, 357–359 (2012). 22388286 10.1038/nmeth.1923 PMC3322381

[76] Quinlan, A. R. & Hall, I. M. BEDTools: a flexible suite of utilities for comparing genomic features. *Bioinformatics* **26**, 841–842 (2010). 20110278 10.1093/bioinformatics/btq033 PMC2832824

[77] Robinson, J. T. et al. Integrative genomics viewer. *Nat. Biotechnol.* **29**, 24 (2011). 21221095 10.1038/nbt.1754 PMC3346182

[78] Machanick, P. & Bailey, T. L. MEME-ChIP: motif analysis of large DNA datasets. *Bioinformatics* **27**, 1696–1697 (2011). 21486936 10.1093/bioinformatics/btr189 PMC3106185

[79] Chuck, G., Whipple, C., Jackson, D. & Hake, S. The maize SBP-box transcription factor encoded by tasselsheath4 regulates bract development and the establishment of meristem boundaries. *Development* **137**, 1243–1250 (2010). 20223762 10.1242/dev.048348

[80] Stitt, M., Lilley, R. M., Gerhardt, R. & Heldt, H. W. Metabolite levels in specific cells and subcellular compartments of plant leaves. In Sidney Fleischer and Becca Fleischer (eds), *Methods in Enzymology*, Vol. 174, 518–552 (Academic Press, Cambridge, Massachusetts, 1989).

