# Supplementary material for: The regulatory landscape of a core maize domestication module controlling bud dormancy and growth repression
Source: Nat Commun. 2019 Aug 23;10:3810. doi: 10.1038/s41467-019-11774-w (PMC6707278; doi:10.1038/s41467-019-11774-w)
Supplement: Supplementary file 1 — Supplementary Information [file 41467_2019_11774_MOESM1_ESM.pdf]

Supplementary Table 1. Expression of *tb1* and *gt1* in tiller buds of B73, *gt1* and *tb1* at 8 and 12 DAP (days after planting).

		[a]samples						[b]Fold change			
**Gene**	**Gene ID**	**B8**	**B12**	**gt8**	**gt12**	**tb8**	**tb12**	**B8/gt8**	**B12/gt12**	**B8/tb8**	**B12/tb**
tb1	AC233950.1_FG002	128.37	120.94	125.92	155.96	24.43	40.00	0.02	-0.22	4.26	2.02
gt1	GRMZM2G005624	319.26	1124.63	294.60	568.14	8.58	12.12	0.08	0.98	36.19	91.81

[a]Samples are tiller buds from B73 developmental series, *gt1* and *tb1*, and show individual normalized CPM (count-per-million) value based on edgeR output. B8, B10, B12 and B14 represent B73 tiller buds at 8, 10, 12 and 14 DAP, respectively; similarly tb8, tb12, gt8, and gt12 stand for *tb1* and *gt1* tiller buds at 8 and 12 DAP, respectively. [b]Fold-change contains the expression ratios of *tb1* or *gt1* between samples: B8/gt8 and B12/gt12 refer to the comparison between B73 and *gt1* at 8 DAP and 12 DAP, respectively; B8/tb8 and B12/tb12 stand for the comparison between B73 and *tb1* at 8 DAP and 12 DAP, respectively.

Supplementary Table 2. Summary statistics for RNA-sequencing and mapping

Lines	Developmental stage	Replicates	Replicate name	Total PE reads (125bp)	Total base pairs (bp)	[a]Total mapped reads(%)	Unique match(%)
B73	8 DAP	-1	B8.1	51601358	12126319130	87.96	82.72
B73	8 DAP	-2	B8.2	37957603	8920036705	87.46	82.02
B73	8 DAP	-3	B8.3	15934498	3776476026	86.08	80.43
B73	10 DAP	-1	B10.1	36261534	8593983558	82.65	76.85
B73	10 DAP	-2	B10.2	34415629	7984425928	84.72	78.67
B73	10 DAP	-3	B10.3	35724740	8466763380	85.32	79.98
B73	12 DAP	-1	B12.1	37344970	8850757890	91.73	86.89
B73	12 DAP	-2	B12.2	30598178	7098777296	86.65	80.72
B73	12 DAP	-3	B12.3	68166498	15814627536	87.97	82.3
B73	14 DAP	-1	B14.1	38995880	9164031800	91.99	86.79
B73	14 DAP	-2	B14.2	39335341	9204469794	87	81.12
B73	14 DAP	-3	B14.3	38287066	8997460510	84.42	78.07
gt1-1	8 DAP	-1	gt8.1	35632000	8373520000	91.6	86.44
gt1-1	8 DAP	-2	gt8.2	59269982	13691365842	90.95	85.87
gt1-1	8 DAP	-3	gt8.3	29277052	6792276064	92.11	87.26
gt1-1	8 DAP	-4	gt8.4	23298364	5405220448	92.14	87.33
gt1-1	8 DAP	-5	gt8.5	37910754	8871116436	90.75	85.81
gt1-1	12 DAP	-1	gt12.1	36250642	8591402154	85.31	79.52
gt1-1	12 DAP	-2	gt12.2	23036949	5759237250	94.87	81.99
gt1-1	12 DAP	-3	gt12.3	19727423	4931855750	94.96	82.87
gt1-1	12 DAP	-4	gt12.4	20982469	5245617250	95.32	83.91
tb1-ref	8 DAP	-1	tb8.1	40581207	9536583645	83.1	77.06
tb1-ref	8 DAP	-2	tb8.2	16641917	3944134329	90.15	85.33
tb1-ref	8 DAP	-3	tb8.3	36555423	8590524405	94.36	90
tb1-ref	12 DAP	-1	tb12.1	19799254	4949813500	95.26	82.52
tb1-ref	12 DAP	-2	tb12.2	18765696	4691424000	95.6	84.81
tb1-ref	12 DAP	-3	tb12.3	20811935	5202983750	95.38	83.4

[a]125bp Pair-end(PE) reads were mapped to the maize reference genome AGPv3 using STAR with default parameter settings.

Supplementary Table 3. List of primers used in this study.

Primer Name	Forward	Reverse	Application
tb1-r genotyping	GCCTTGGAGTCCCATCAGTA	TTCATCGTCACACAGCCAAT	tb1-r mutant genotyping
gt1 expression	GAGCTCAGCTTCAGGAAGGA	CATGAGCTTGCTCTTGTGG	gt1 gene expression by RT-qPCR
gt1_p1	TCTCATCGACTGTTGACGACGG	GGCCGTAGAAAGACGATGCAAA	ChIP-qPCR
gt1_p2	GCTTGAAAGGAAAGGACCGGTG	CACACAGCTACTGTTCGAGGGA	ChIP-qPCR
gt1_p3	GCGGCGCAATAAGAGTAAGA	CAAACCTCCACACTCCGAAT	ChIP-qPCR
gt1_p4	ACCGAAAAGGGTCAACTGTG	CAGCCGTGAGTGTGAGAGAG	ChIP-qPCR
gt1_p5	CCCAAGTAGGGGTACAGCAA	CTACTTGGATCGGTCGTCGT	ChIP-qPCR
gt1_p6	TGCTTCTTGTTGTCCTGTGC	AGAGAAAAGGCGTGGAGTGA	ChIP-qPCR
gt1_p7	TGTTGACACCGCAGGAATTA	TCATCTCCTTTCCCACCATC	ChIP-qPCR
tga1_p1	ACAGGTGCACAGCACAACAT	AGGAGCGTGTGCATGAAAAG	ChIP-qPCR
tga1_p2	GCTTGCTTTTCCGAGCGGTT	GGAGTACACCCCTGTCTCGC	ChIP-qPCR

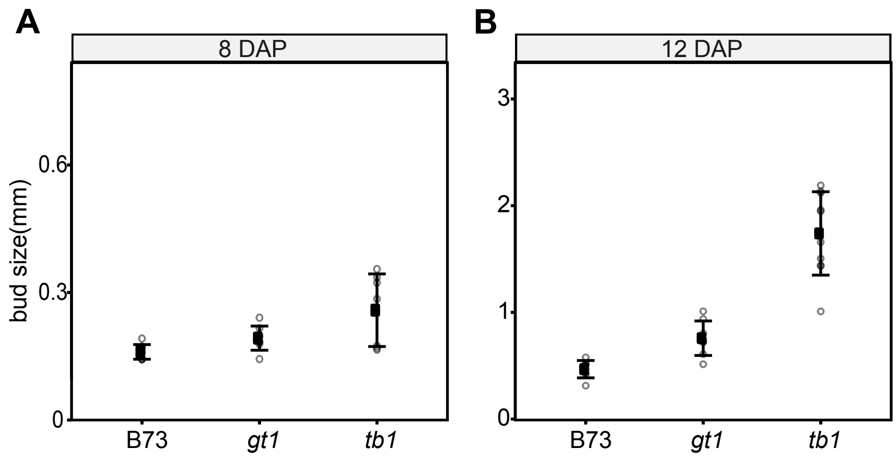

Supplementary Fig. 1. Length of tiller buds in the first (L1) leaf axis of B73, *gt1* and *tb1* at 8 (A) and 12 (B) days after planting (DAP). Bars for each stage represent the mean ± standard error of 8 replicates.

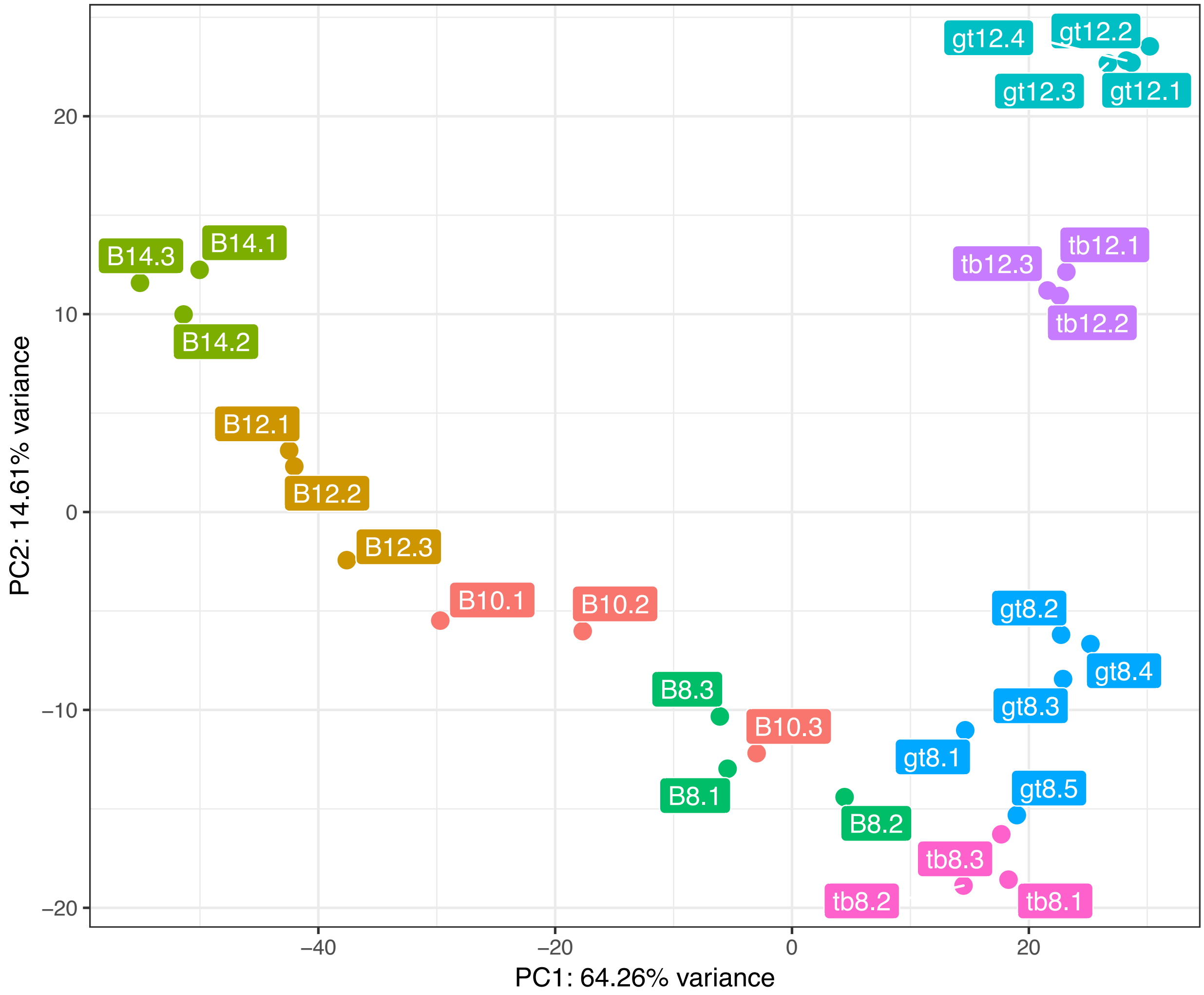

Supplementary Fig. 2. Principal component analysis of 27 RNA-seq libraries collected from the tiller buds of B73 developmental series, *gt1* and *tb1*. Principal component (PC) 1 and PC2 are graphically visualized. B8, B10, B12 and B14 represent B73 tiller buds at 8, 10, 12 and 14 DAP, respectively; similarly tb8, tb12, gt8, and gt12 stand for *tb1* and *gt1* tiller buds at 8 and 12 DAP, respectively. Colored dots represent individual biological replicates. Each biological replicate was specified with the corresponding sample name following by a dot symbol and a unique numeric number.

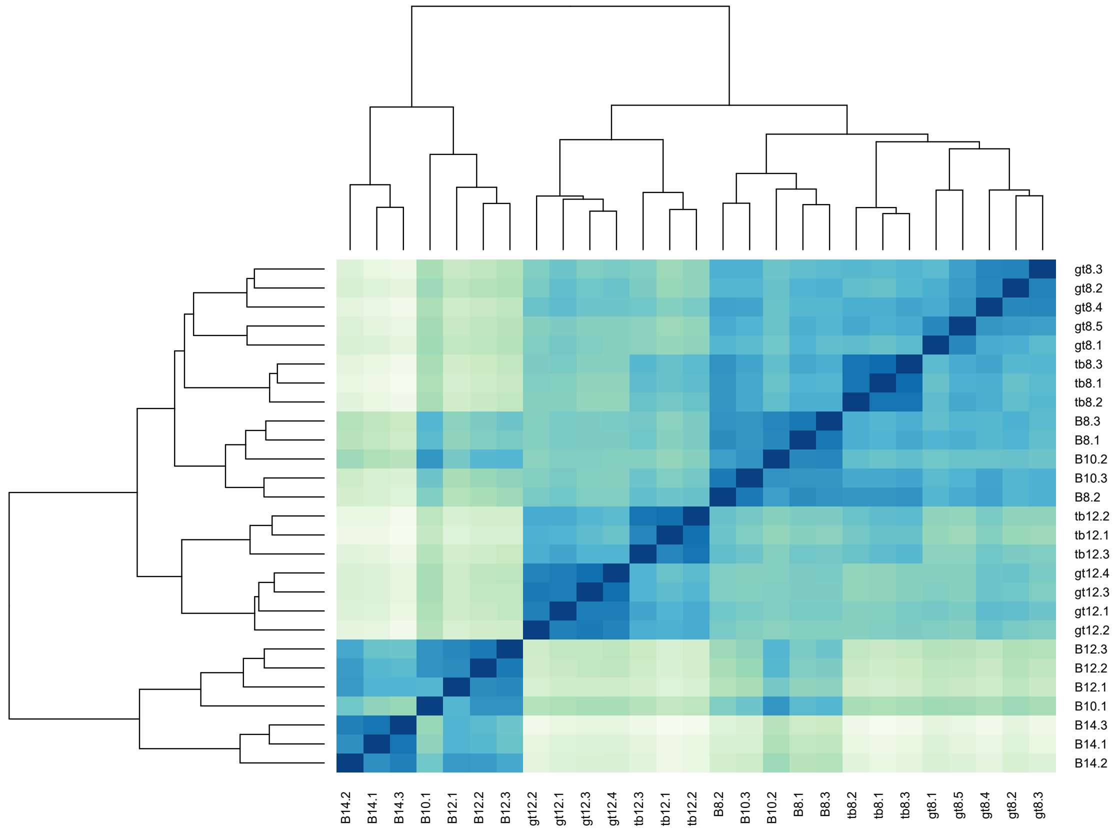

Supplementary Fig. 3. Hierarchical clustering of 27 RNA-seq libraries collected from the tiller buds of B73 developmental series, *gt1* and *tb1*. B8, B10, B12 and B14 represent B73 tiller buds at 8, 10, 12 and 14 DAP, respectively; similarly tb8, tb12, gt8, and gt12 stand for *tb1* and *gt1* tiller buds at 8 and 12 DAP, respectively. Each biological replicate was specified with the corresponding sample name following by a dot symbol and a unique numeric number.

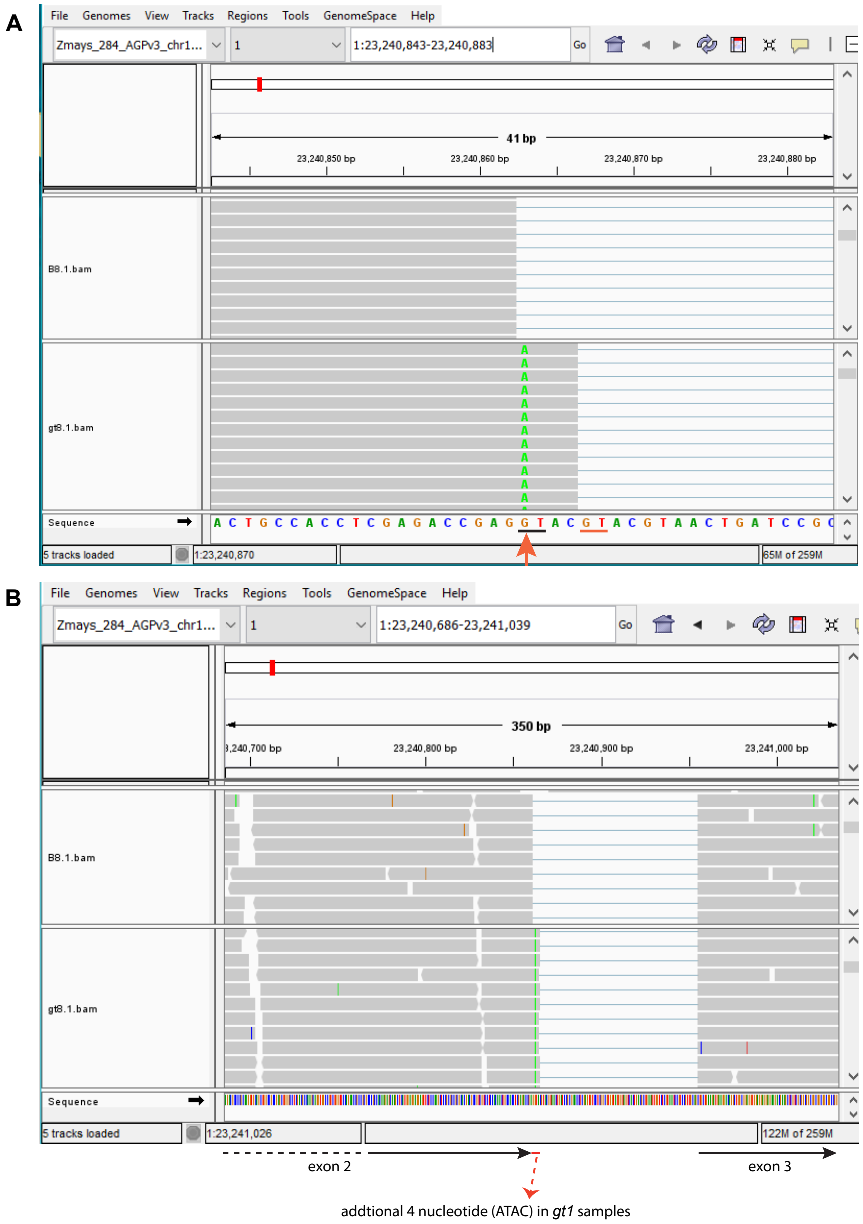

Supplementary Fig. 4. *gt1* mutation resulted in the accumulation of frameshifted *gt1* transcripts in *gt1* mutant. **(A-B)** screencaps showing the mapping of RNA-seq raw reads from tiller buds of B73 and *gt1* to the *gt1* genomic region of maize B73 reference genome. B8.1 and gt8.1 stand for one of the biological replicates of B73 and *gt1* at 8DAP, respectively. B12.1 and gt12.1 refer for one of the biological replicates of B73 and *gt1* at 12 DAP, respectively. (A) G>A mutation was detected in the splicing region (underlines in black) of exon 2 in *gt1* samples. (B) Such mutation abolished the original splice donor site and resulted in a four-nucleotide (ATAC) insertion (underlines in red) in *gt1* mRNA which subsequently caused the accumulation of frameshifted and non-functional *gt1* transcripts in *gt1* mutant.

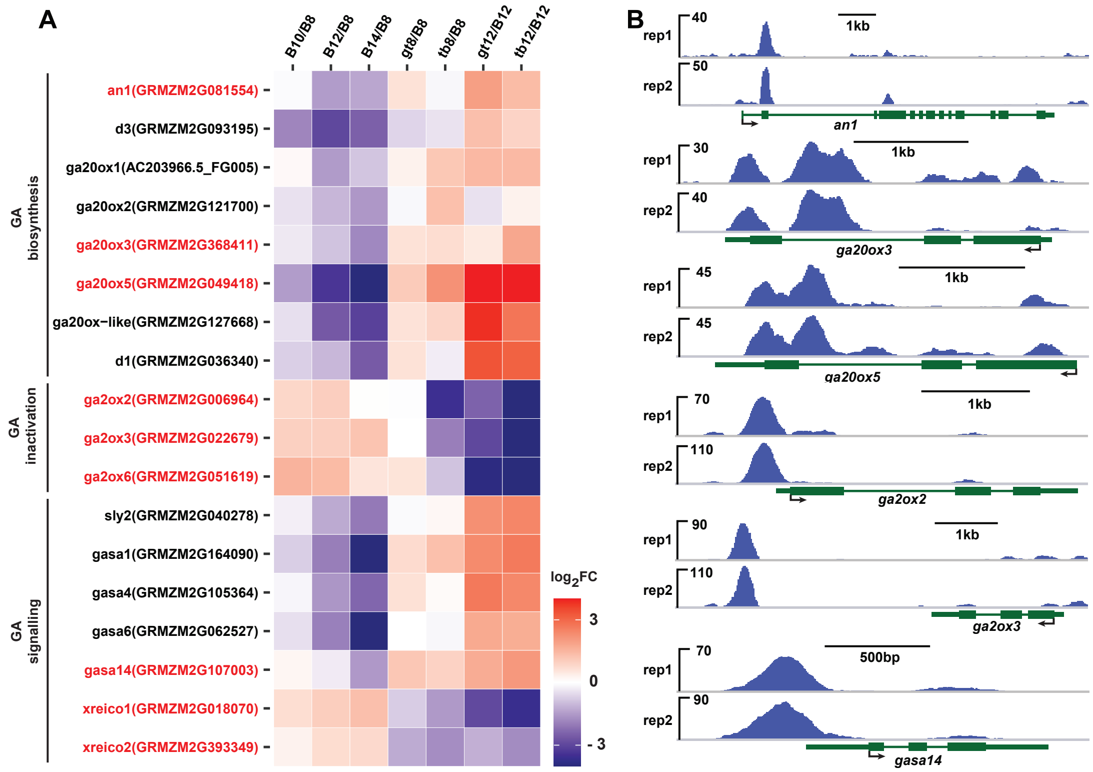

Supplementary Fig. 5. Bud outgrowth suppression is associated with *tb1-gt1* mediated regulation of GA biosynthesis, GA inactivation and GA signaling. (**A**) Genes involved in GA biosynthesis, GA inactivation and GA signaling were differentially expressed in tiller buds across B73 developmental series, *gt1* and *tb1*. B8, B10, B12 and B14 represent B73 tiller buds at 8, 10, 12 and 14 DAP (days after planting), respectively; similarly tb8, tb12, gt8, and gt12 stand for *tb1* and *gt1* tiller buds at 8 and 12 DAP, respectively. slashes representing pairwise comparison between two samples (e.g. B10/B8 is a pairwise comparison between B73 buds at 10 vs 8 DAP). Gene names and GRMZ ID numbers were highlighted in red if they were also directly targeted by TB1 in ChIP-seq assay. FC, fold change. Gradient color scale indicates the log value of expression fold change (log_2FC). (**B**) TB1 ChIP-Seq binding peaks near differentially expressed GA genes from (A). rep1 and rep2 represent two biological replicates of TB1 ChIP-seq assay.

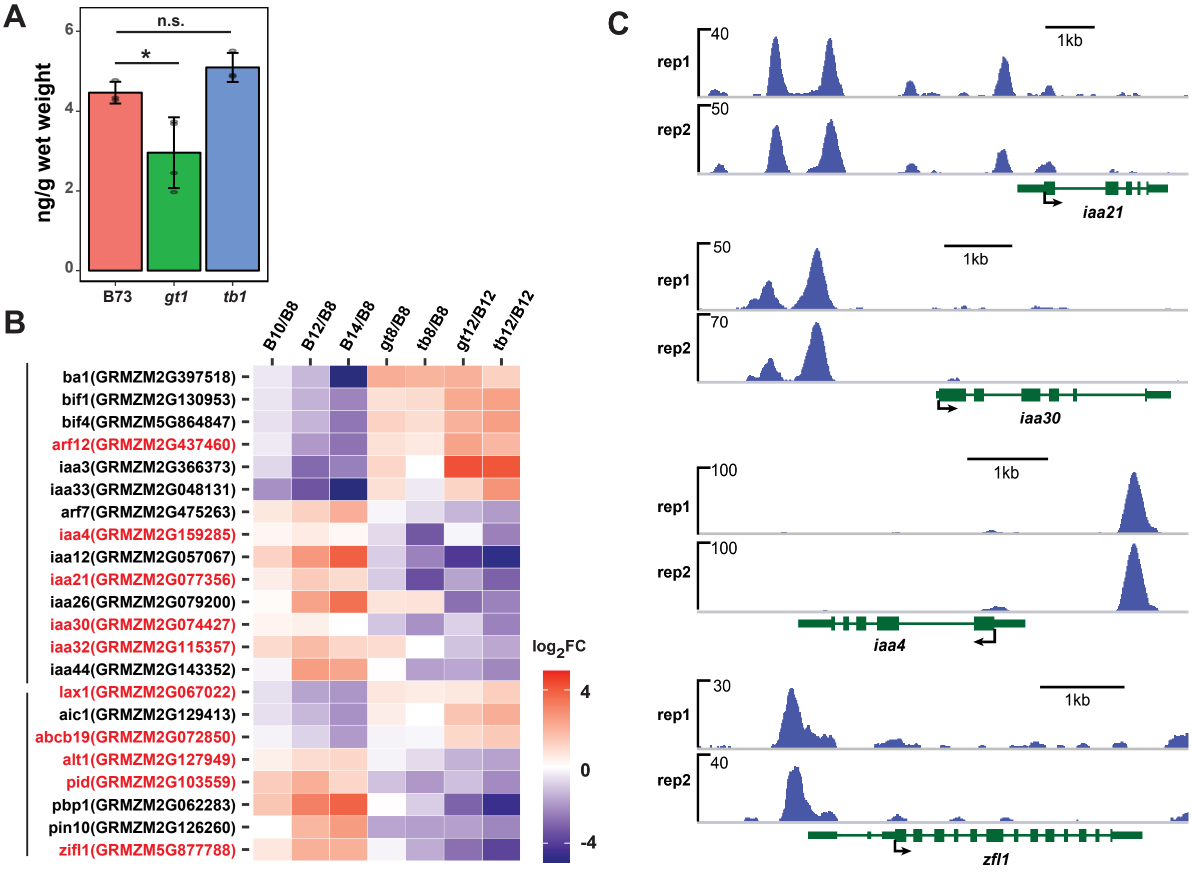

Supplementary Fig. 6. Bud outgrowth suppression is associated with *tb1-gt1* mediated regulation of auxin signaling and auxin distribution in tiller buds. (A) Quantification of IAA levels in tiller buds of B73, *gt1* and *tb1*. Data are means ± SE calculated from at least three biological replicates. n.s. not significant difference detected. *p < 0.05, 2-tailed Student's t test. (B) Genes involved in auxin signaling and auxin distribution were differentially expressed in tiller buds across B73 developmental series, *gt1* and *tb1*. B8, B10, B12 and B14 represent B73 tiller buds at 8, 10, 12 and 14 DAP (days after planting), respectively; similarly *tb8, tb12, gt8,* and *gt12* stand for *tb1* and *gt1* tiller buds at 8 and 12 DAP, respectively. slashes representing pairwise comparison between two samples (e.g. B10/B8 is a pairwise comparison between B73 buds at 10 vs 8 DAP). Gene names and GRMZM ID numbers were highlighted in red if they were also directly targeted by TB1 in ChIP-seq assay. FC, fold change. Gradient color scale indicates the log value of expression fold change (log2FC). (C) TB1 ChIP-Seq binding peaks near differentially expressed auxin genes from (B). rep1 and rep2 represent two biological replicates of TB1 ChIP-seq assay.

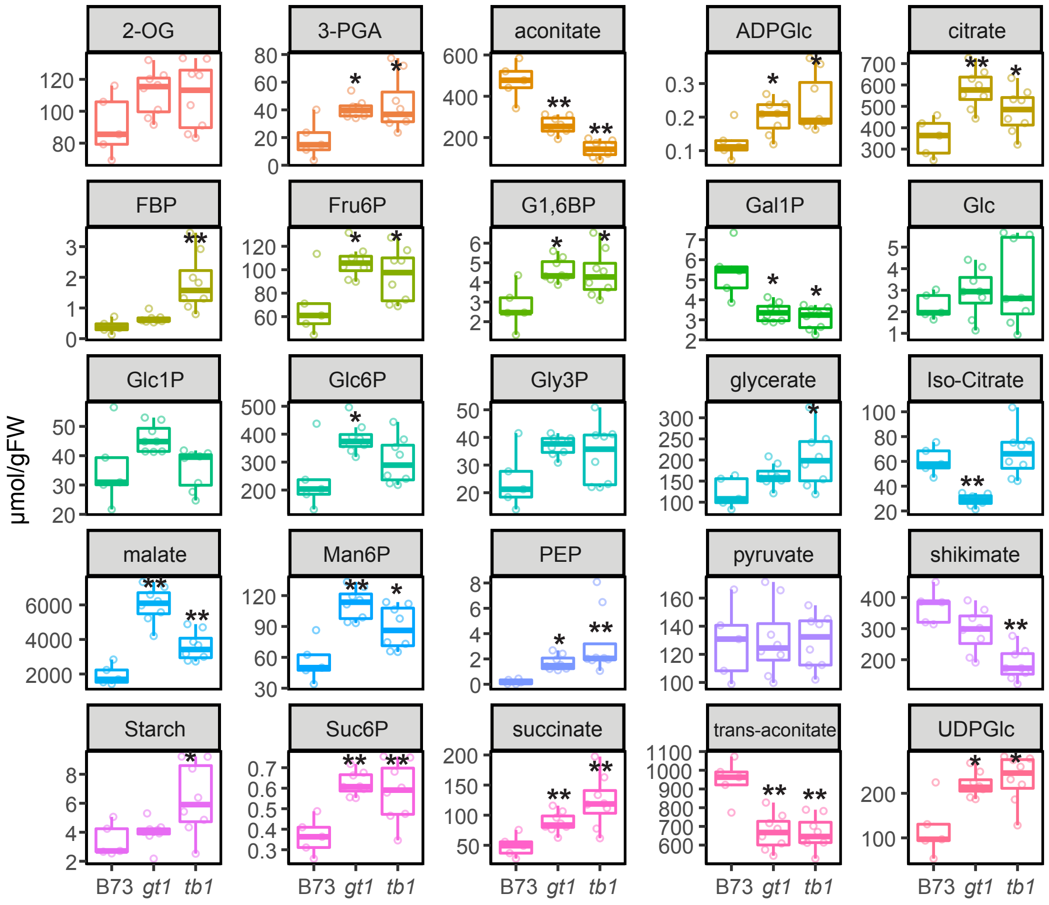

Supplementary Fig. 7. Quantification of carbohydrates levels in tiller buds of B73, *gt1* and *tb1*. Plots show means ± SE calculated from at least five biological replicates. Significant analysis (2-tailed Student's t test) was carried out to evaluate the difference between B73 and *gt1* or between B73 and *tb1*. *$p < 0.05$, **$p < 0.01$. 2-OG, 2-oxoglutarate; 3-PGA,3-Phosphoglyceric acid; ADPGlc, adenosine diphosphoglucose; FBP, fructose 1,6-bisphosphate; Fru6P, fructose-6-phosphate; G1,6BP, glucose 1,6-bisphosphate; Gal1P, galactose-1-phosphate; Glc, glucose; Glc1P, glucose-1-phosphate; Glc6P, glucose-6-phosphate; Gly3P, glycerol 3-phosphate; Man6P, Mannose-6-phosphate; PEP, phosphoenolpyruvate; Suc6P, sucrose-6-phosphate; UDPGlc, uridine diphosphoglucose.

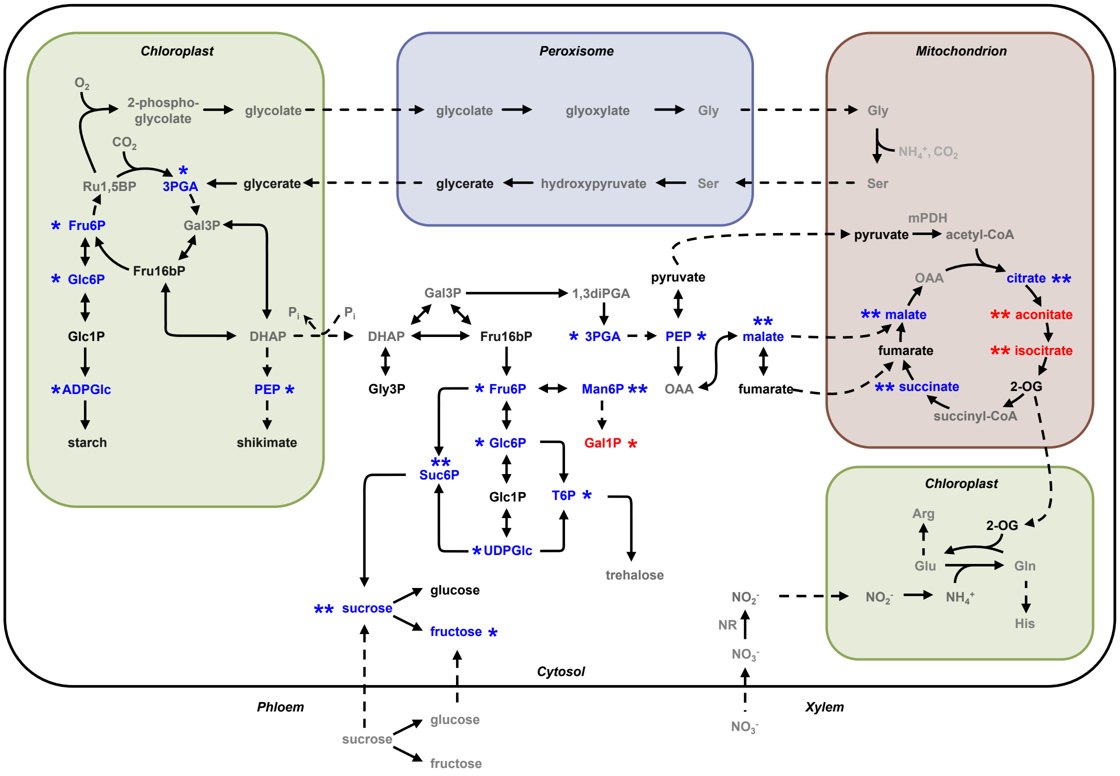

Supplementary Fig. 8. Overview of carbohydrate changes in *gt1* buds compared to B73 buds.

Carbohydrate metabolite data from Fig.4 and Fig. S7 are displayed on a map of central metabolism that was adapted from Figueroa (Figueroa et al. 2016). Solid arrows represent single-enzyme reactions and dashed arrows represent multiple reactions or transport between compartments. Significant analysis (2-tailed Student's t test) was carried out to evaluate the difference between B73 and *gt1*. *p < 0.05, **p < 0.01. Colors indicate which metabolites were significantly increased (blue) or decreased (red), or unchanged (black). Metabolites shown in grey were not measured. Suc, sucrose; Fru, fructose; Glc, glucose; Suc6P,sucrose-6-phosphate; Fru6P,fructose-6-phosphate; Glc6P,glucose-6-phosphate; T6P,trehalose-6-phosphate; Fru16bP, fructose 1,6-bisphosphate; UDPGlc, uridine diphosphoglucose; Man6P,Mannose-6-phosphate; Gly3P,glycerol 3-phosphate; 3-PGA,3-Phosphoglyceric acid; Glc1P,glucose-1-phosphate; ADPGlc, adenosine diphosphoglucose; PEP, phosphoenolpyruvate; Gal1P,galactose-1-phosphate; 2-OG,2-oxoglutarate.

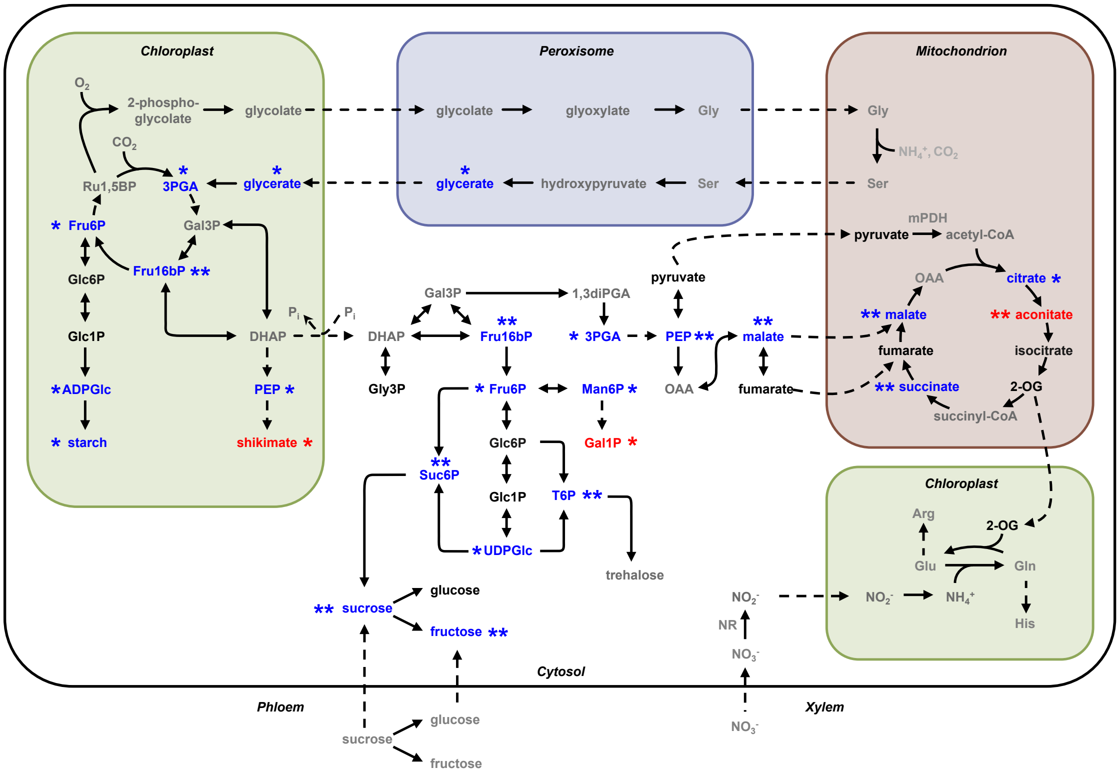

Supplementary Fig. 9. Overview of carbohydrate changes in *tb1* buds compared to B73 buds.

Carbohydrate metabolite data from Fig.4 and Fig. S7 are displayed on a map of central metabolism that was adapted from Figueroa (Figueroa et al. 2016). Solid arrows represent single-enzyme reactions and dashed arrows represent multiple reactions or transport between compartments. Significant analysis (2-tailed Student's t test) was carried out to evaluate the difference between B73 and *tb1*. *$p < 0.05$, **$p < 0.01$. Colors indicate which metabolites were significantly increased (blue) or decreased (red), or unchanged (black). Metabolites shown in grey were not measured. Suc, sucrose; Fru, fructose; Glc, glucose; Suc6P, sucrose-6-phosphate; Fru6P, fructose-6-phosphate; Glc6P,glucose-6-phosphate; T6P, trehalose-6-phosphate; Fru16bP, fructose 1,6-bisphosphate; UDPGlc, uridine diphosphoglucose; Man6P,Mannose-6-phosphate; Gly3P,glycerol 3-phosphate; 3-PGA,3-Phosphoglyceric acid; Glc1P,glucose-1-phosphate; ADPGlc, adenosine diphosphoglucose; PEP, phosphoenolpyruvate; Gal1P,galactose-1-phosphate; 2-OG,2-oxoglutarate.

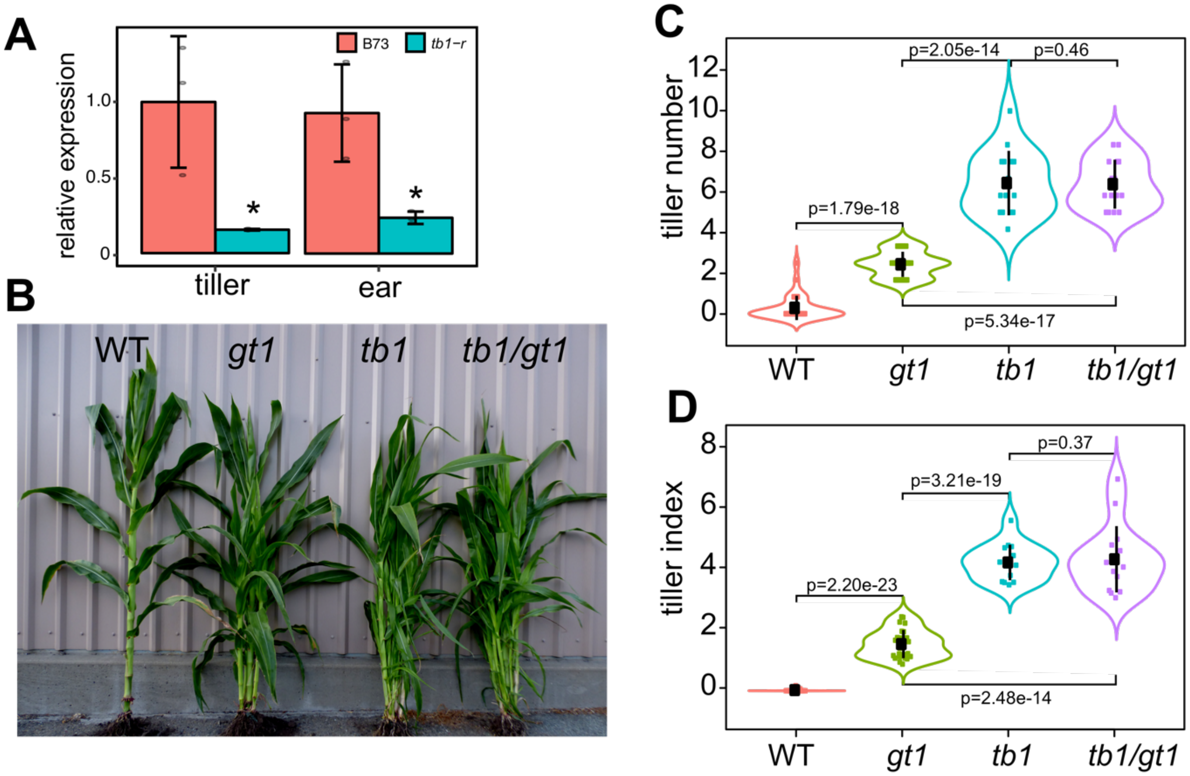

Supplementary Fig. 10. *gt1* is genetically downstream of *tb1*.

(A) qRT-PCR revealed the transcript of *gt1* is down-regulated in *tb1-r* mutant, consistent with the finding by RNA-seq. * represents statistical significance with $p < 0.001$ by t-test. (B) Double mutant of *tb1/gt1* analysis using siblings from the same segregating family within B73 background. (C-D) Statistical comparison of the double mutant phenotype in terms of tiller number (C) and tiller index (calculated by the ratio of the sum of tiller lengths to the height of the plant, D). p value was calculated by One-Way ANOVA Test.
