# Supplementary material for: The regulatory landscape of a core maize domestication module controlling bud dormancy and growth repression
Source: Nat Commun. 2019 Aug 23;10:3810. doi: 10.1038/s41467-019-11774-w (PMC6707278; doi:10.1038/s41467-019-11774-w)
Supplement: Supplementary file 2 — Description of Additional Supplementary Files [file 41467_2019_11774_MOESM2_ESM.pdf]

Description of Additional Supplementary Files

File Name: Supplementary Data 1
Description: Genes expressed in tiller buds across B73 developmental series, gt1 and tb1.

File Name: Supplementary Data 2
Description: Genes expressed in tiller buds across B73 developmental series, gt1 and tb1.

File Name: Supplementary Data 3
Description: Genes differentially expressed in tiller buds across B73 developmental series.

File Name: Supplementary Data 4
Description: Genes differentially expressed in tiller buds between B73 and the two tillering mutants (tb1 and gt1) at 8 and 12 DAP (days after planting).

File Name: Supplementary Data 5
Description: Genes differentially expressed both across B73 developmental series and between B73 and the two tillering mutants (tb1 and gt1).

File Name: Supplementary Data 6
Description: Dynamically expressed genes across B73 developmental series, gt1 and tb1 were grouped by K-Means clustering method in coseq package.

File Name: Supplementary Data 7
Description: Genes differentially expressed between B73 and tb1 at 8 DAP (days after planting).

File Name: Supplementary Data 8
Description: Genes differentially expressed between B73 and tb1 at 12 DAP (days after planting).

File Name: Supplementary Data 9
Description: Genes differentially expressed between B73 and tb1 at both 8 and 12 DAP (days after planting).

File Name: Supplementary Data 10
Description: Genes differentially expressed between B73 and gt1 at 8 DAP (days after planting).

File Name: Supplementary Data 11
Description: Genes differentially expressed between B73 and gt1 at 12 DAP (days after planting).

File Name: Supplementary Data 12
Description: Genes differentially expressed between B73 and gt1 at both 8 and 12 DAP (days after planting).

File Name: Supplementary Data 13
Description: Shared differentially expressed genes between tb1 vs. B73 and gt1 vs. B73 at 8 DAP (days after planting).

File Name: Supplementary Data 14

Description: Shared differentially expressed genes between tb1 vs. B73 and gt1 vs. B73 at 12 DAP (days after planting).

File Name: Supplementary Data 15
Description: Shared differentially expressed genes between tb1 vs. B73 and gt1 vs. B73 at both 8 and 12 DAP (days after planting).

File Name: Supplementary Data 16
Description: TB1 targeted genes by ChIP-seq.

File Name: Supplementary Data 17
Description: Sequence alignment of the TB1 binding region in the tb1, prolificacy locus and tga1 promoter.

File Name: Supplementary Data 18
Description: Genes expressed in tiller buds across B73 developmental series, gt1 and tb1.
