# Supplementary material for: The regulatory landscape of a core maize domestication module controlling bud dormancy and growth repression
Source: Nat Commun. 2019 Aug 23;10:3810. doi: 10.1038/s41467-019-11774-w (PMC6707278; doi:10.1038/s41467-019-11774-w)
Supplement: Supplementary file 19 — Supplementary Data 17 [file 41467_2019_11774_MOESM19_ESM.pdf]

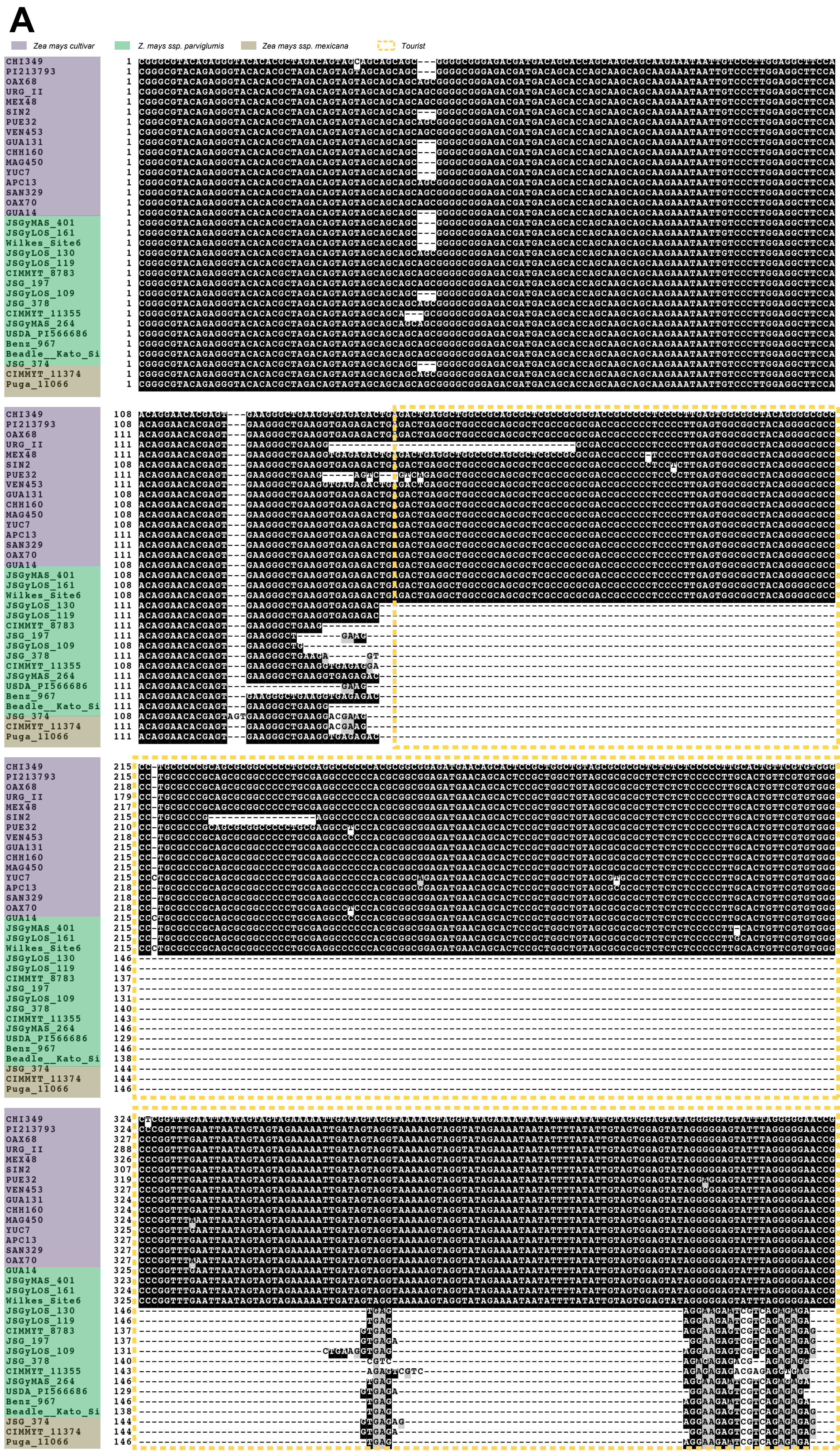
A
Zea mays cultivar
Z. mays ssp. parviglumis
Zea mays ssp. mexicana
Tourist

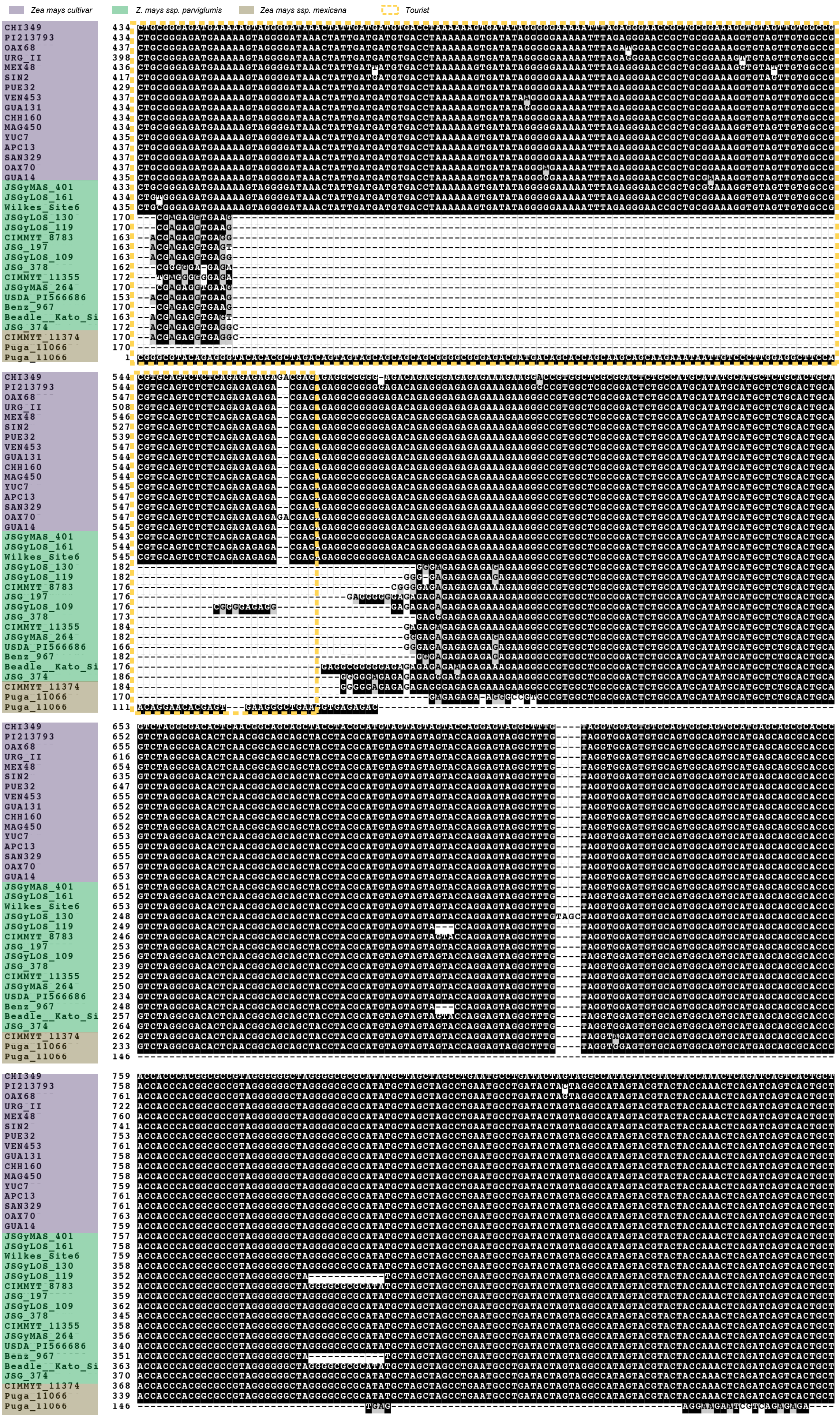

Zea mays cultivar
Z. mays ssp. parviglumis
Zea mays ssp. mexicana
Tourist
CHI349
PI213793
OAX68
URG_II
MEX48
SIN2
PUE32
VEN453
GUA131
CHH160
MAG450
YUC7
APC13
SAN329
OAX70
GUA14
JSGyMAS_401
JSGyLOS_161
Wilkes_Site6
JSGyLOS_130
JSGyLOS_119
CIMMYT_8783
JSG_197
JSGyLOS_109
JSG_378
CIMMYT_11355
JSGyMAS_264
USDA_PI566686
Benz_967
Beadle__Kato_Si
JSG_374
CIMMYT_11374
Puga_11066
Puga_11066

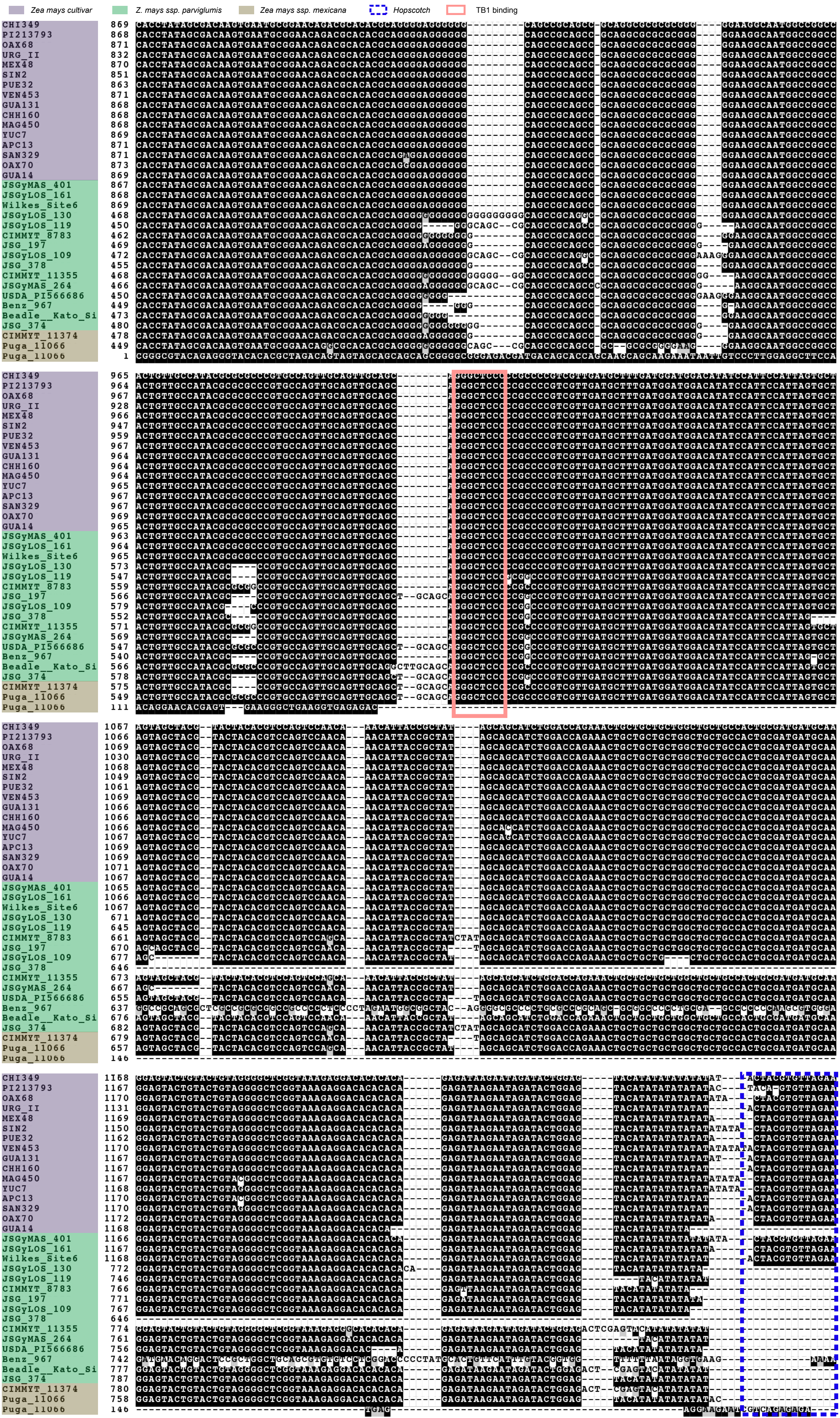
Zea mays cultivar
Z. mays ssp. parviglumis
Zea mays ssp. mexicana
Hopscotch
TB1 binding

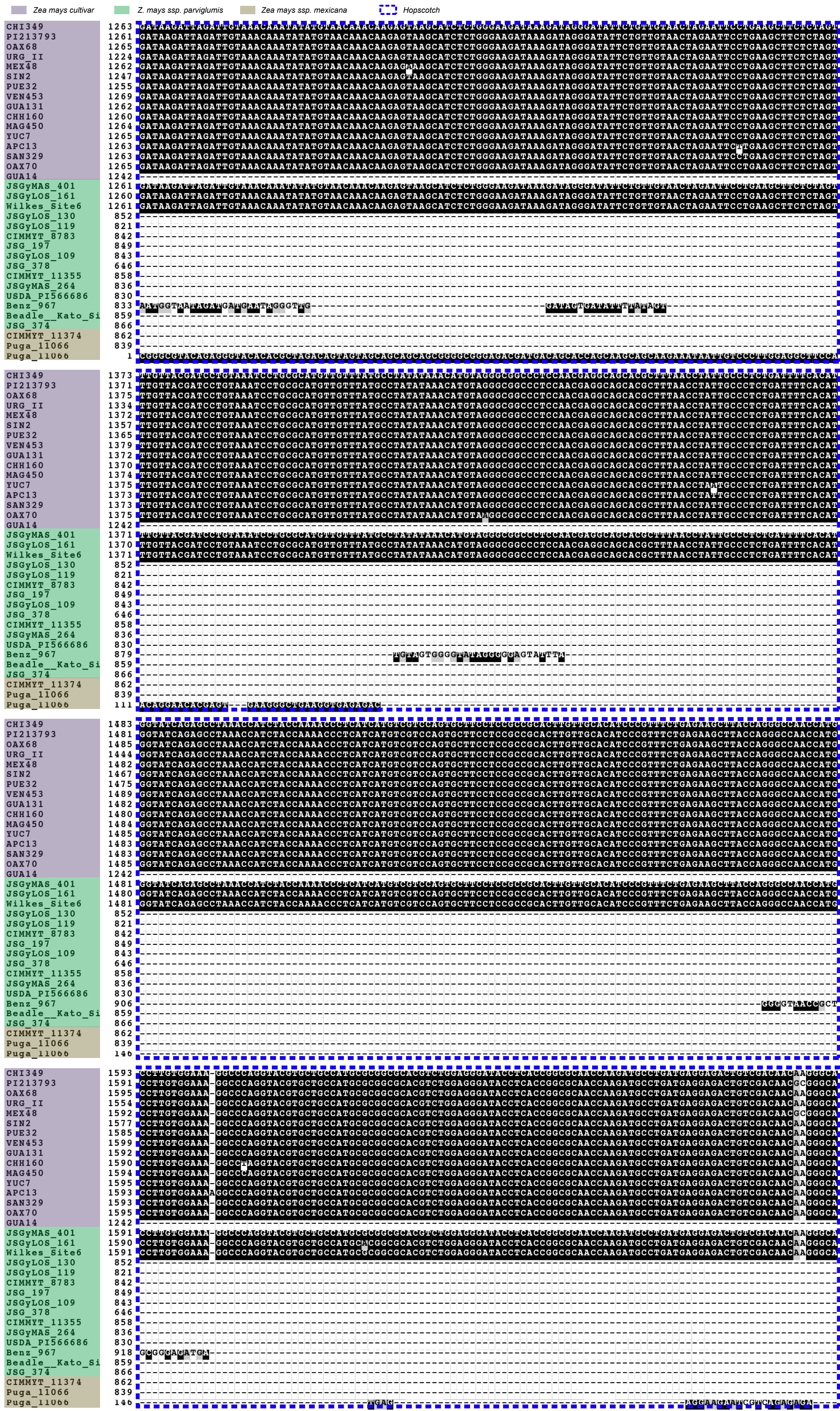
Zea mays cultivar
Z. mays ssp. parviglumis
Zea mays ssp. mexicana
Hopscotch

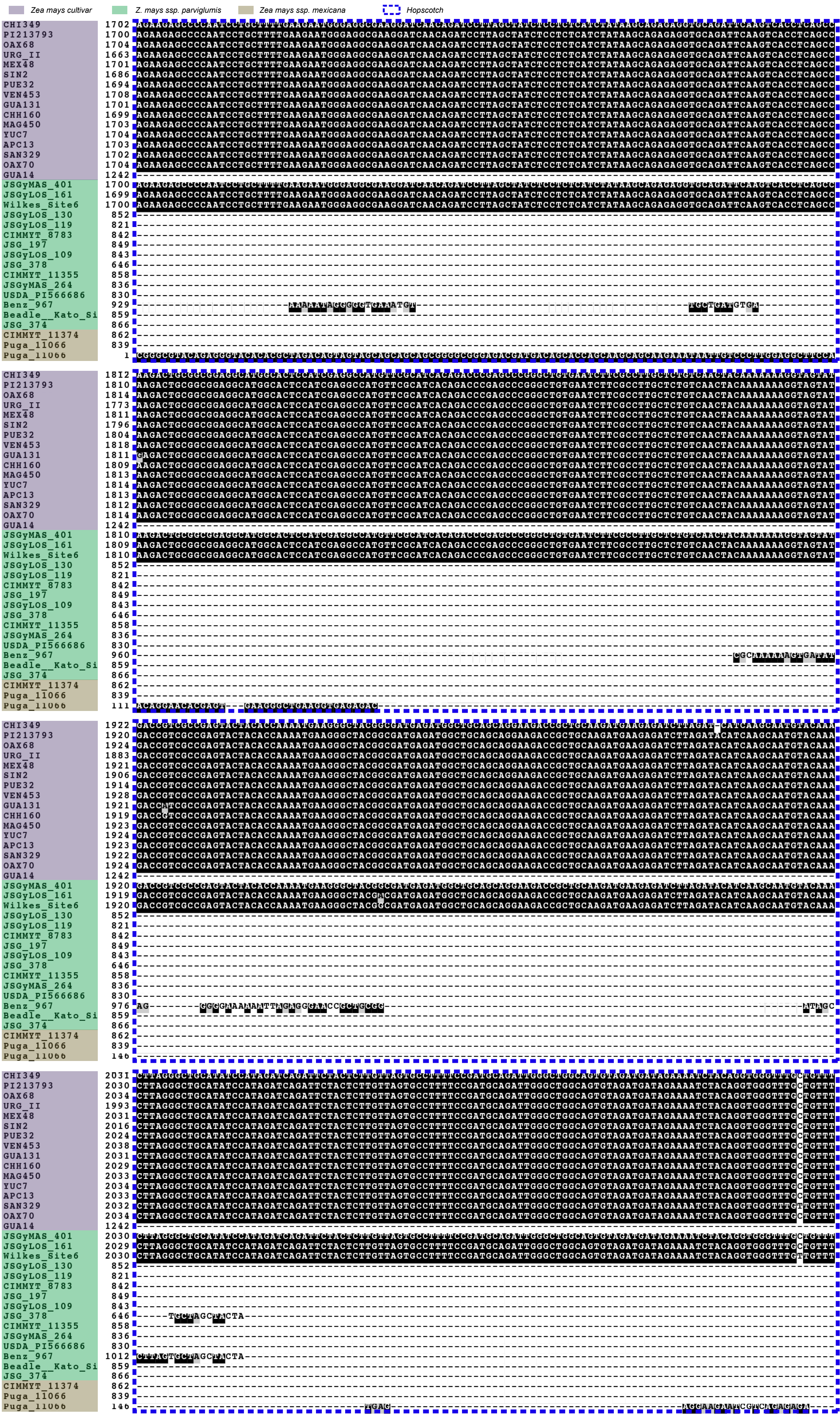
Zea mays cultivar
Z. mays ssp. parviglumis
Zea mays ssp. mexicana
Hopscotch

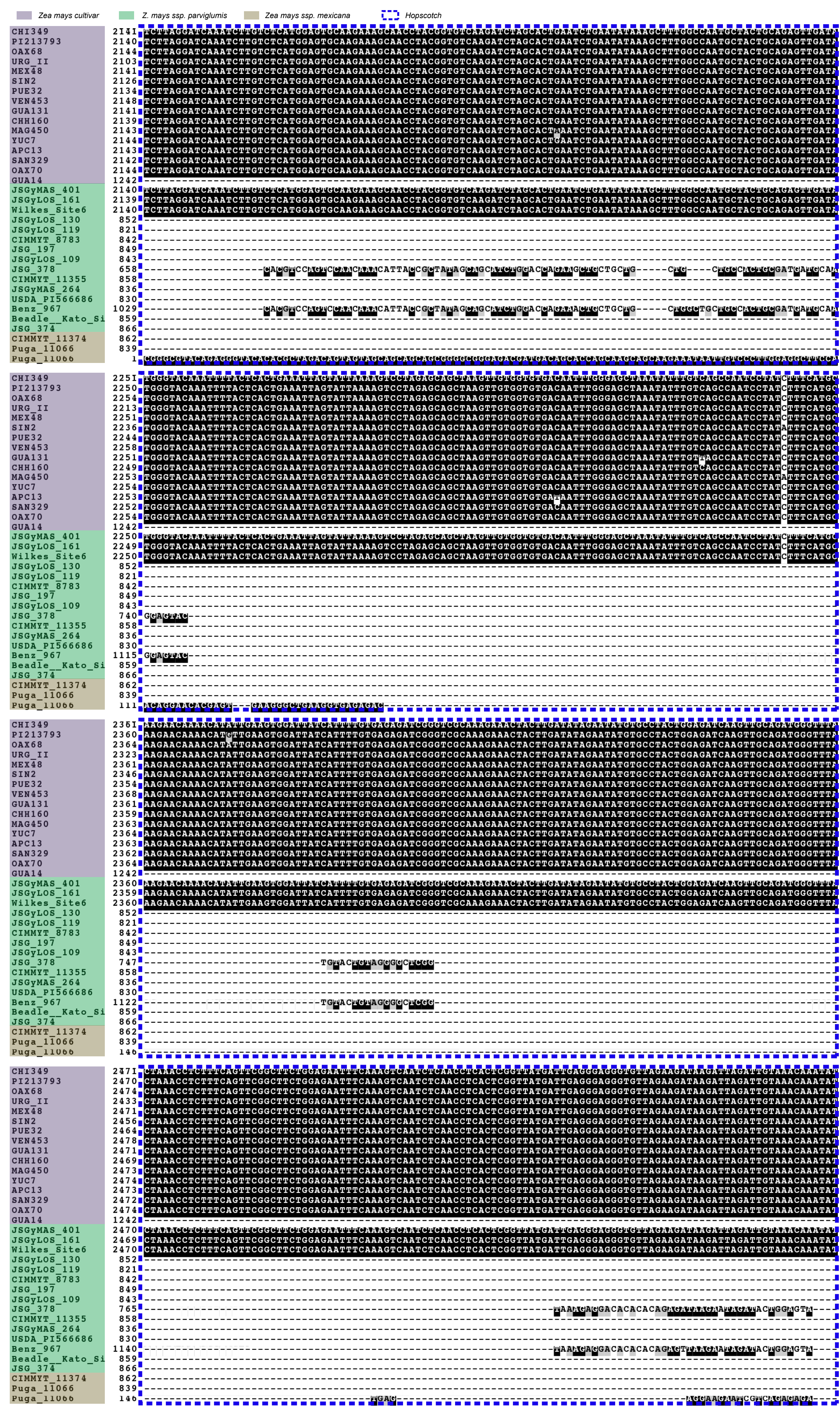
Zea mays cultivar
Z. mays ssp. parviglumis
Zea mays ssp. mexicana
Hopscotch

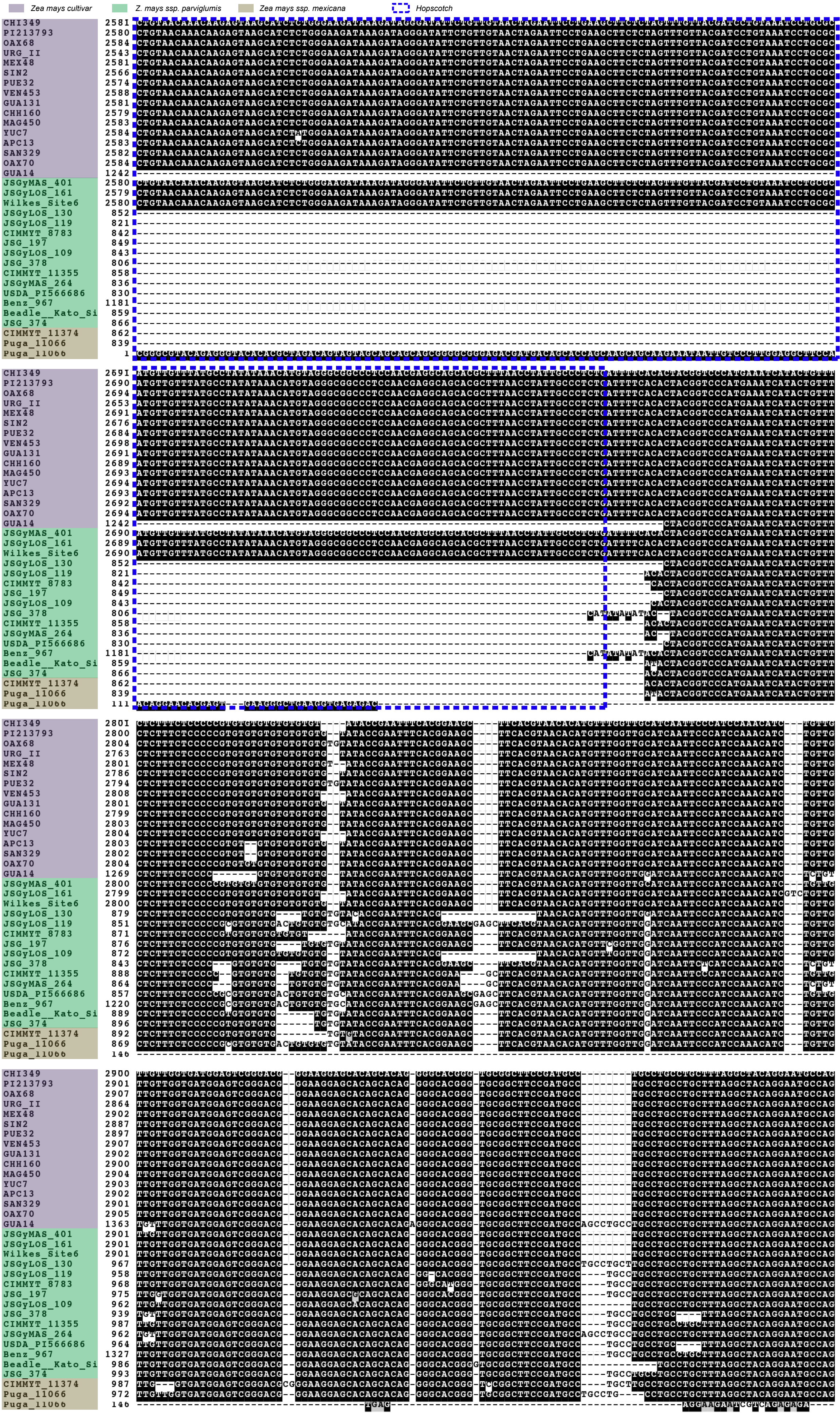
Zea mays cultivar
Z. mays ssp. parviglumis
Zea mays ssp. mexicana
Hopscotch

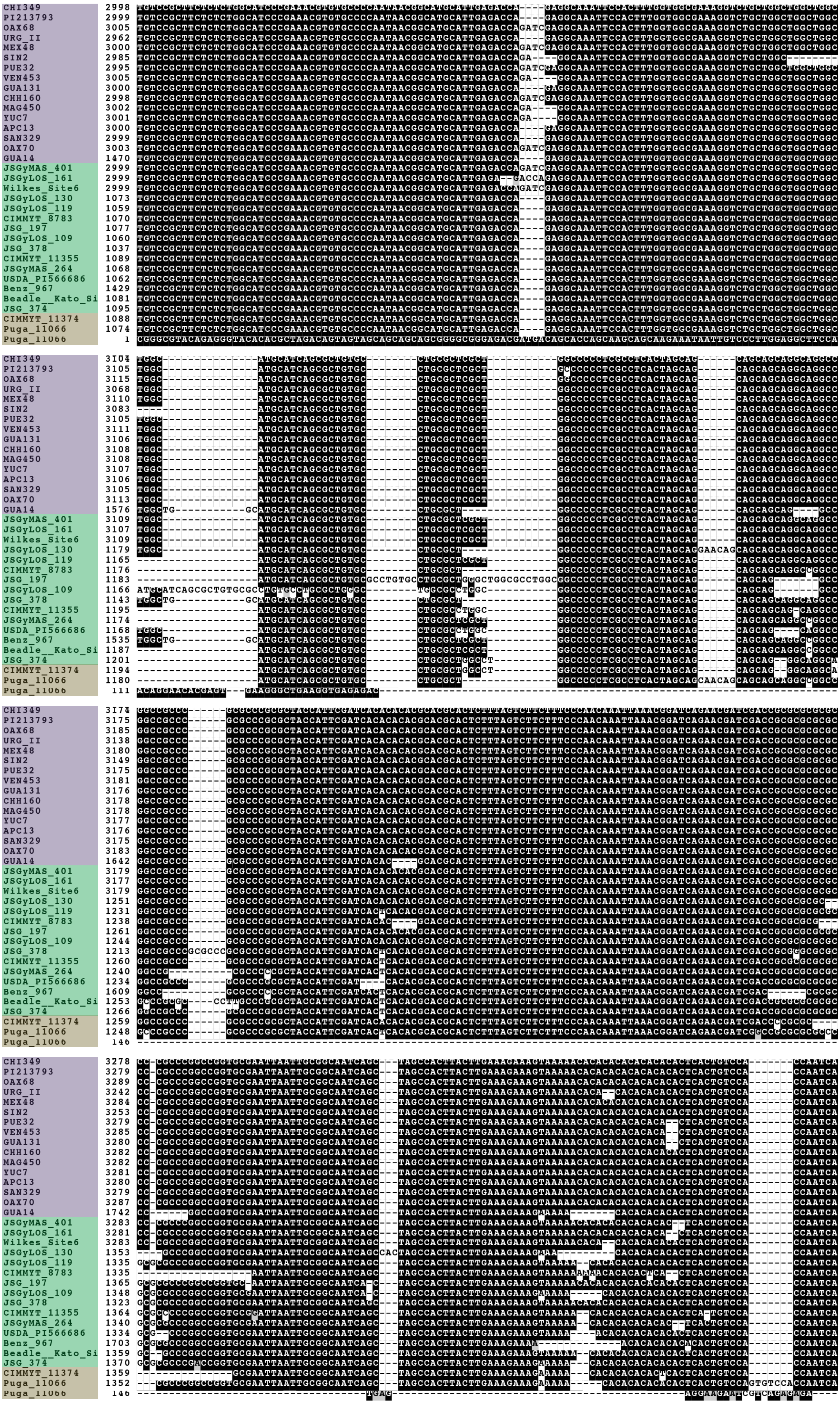
Zea mays cultivar
Z. mays ssp. parviglumis
Zea mays ssp. mexicana

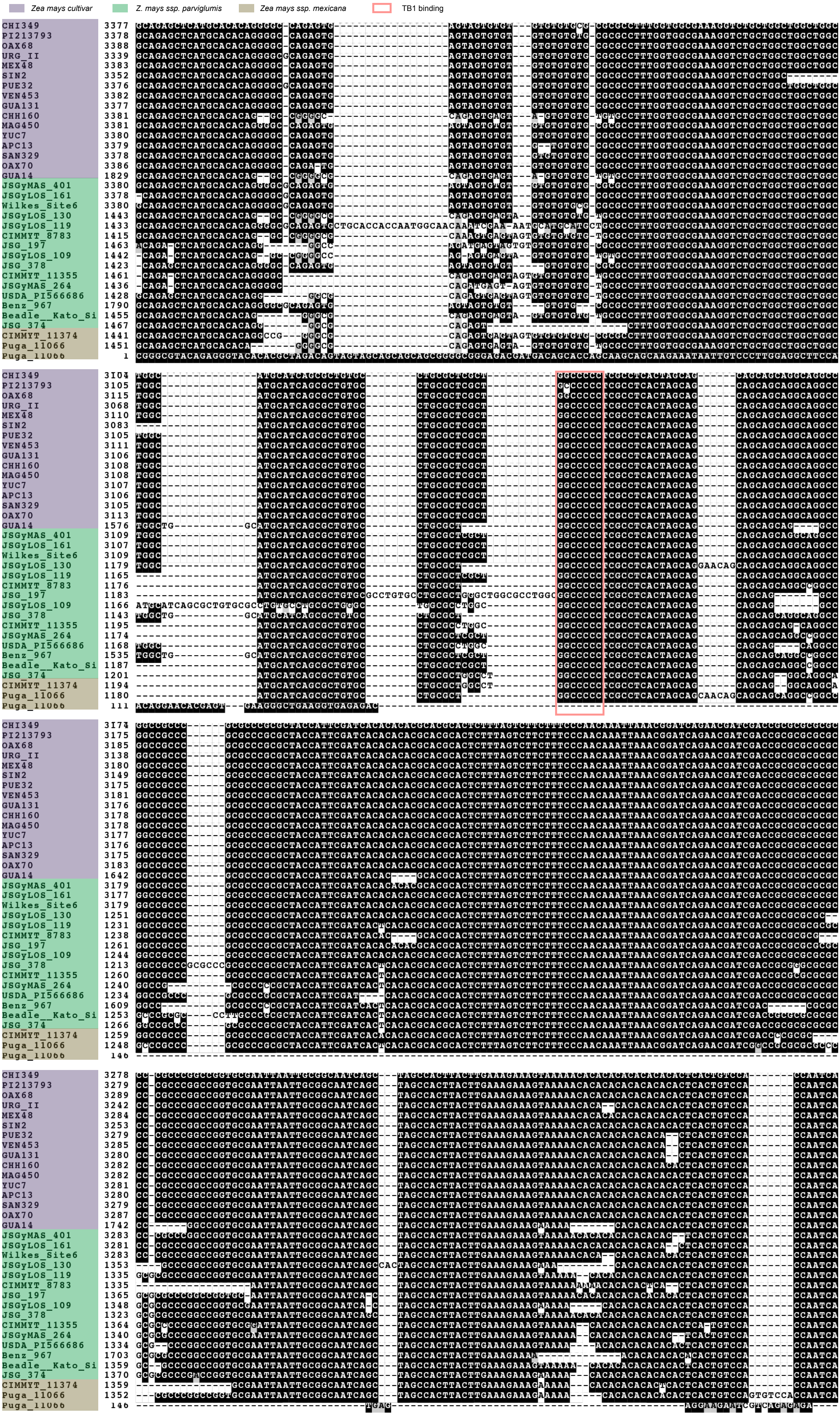

Zea mays cultivar
Z. mays ssp. parviglumis
Zea mays ssp. mexicana
TB1 binding

B

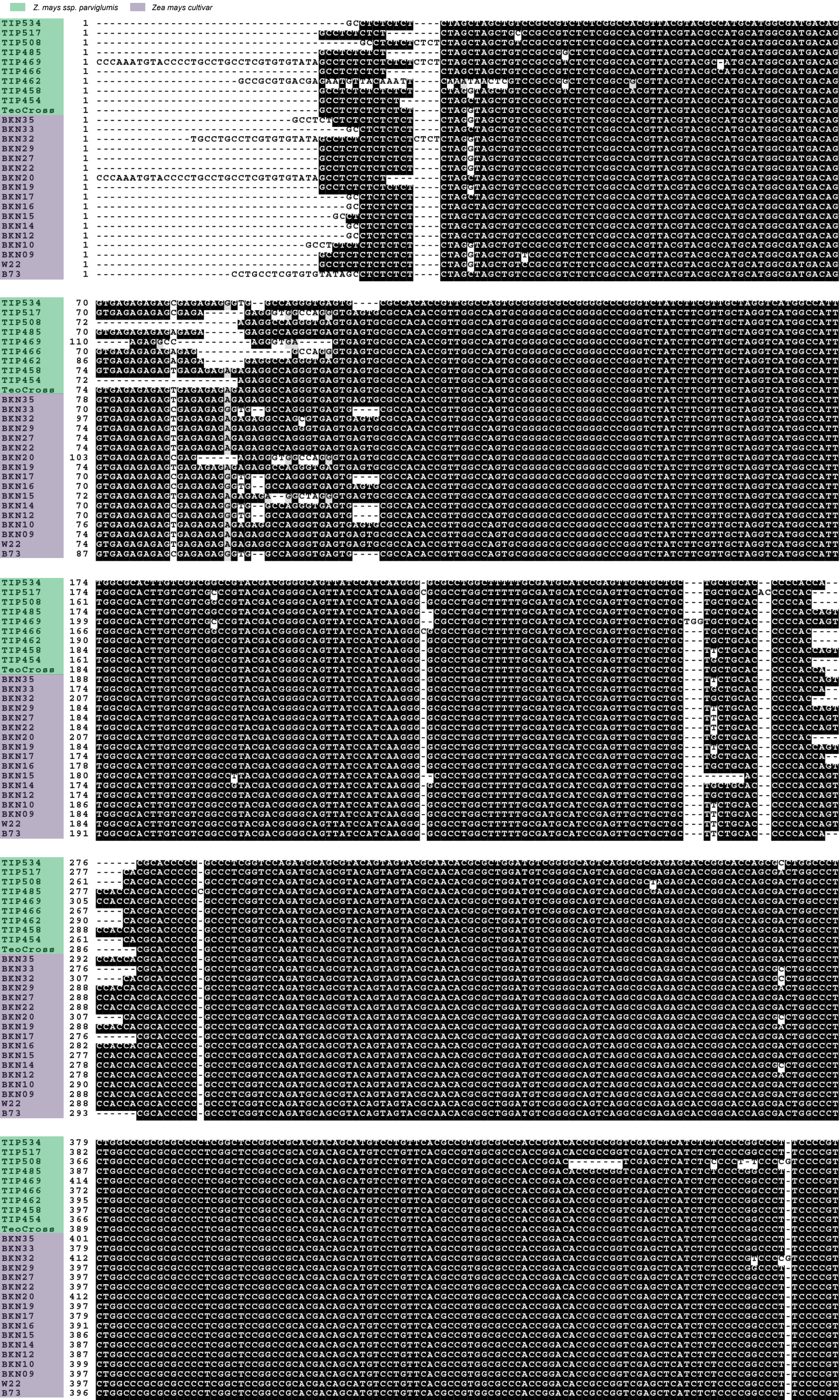

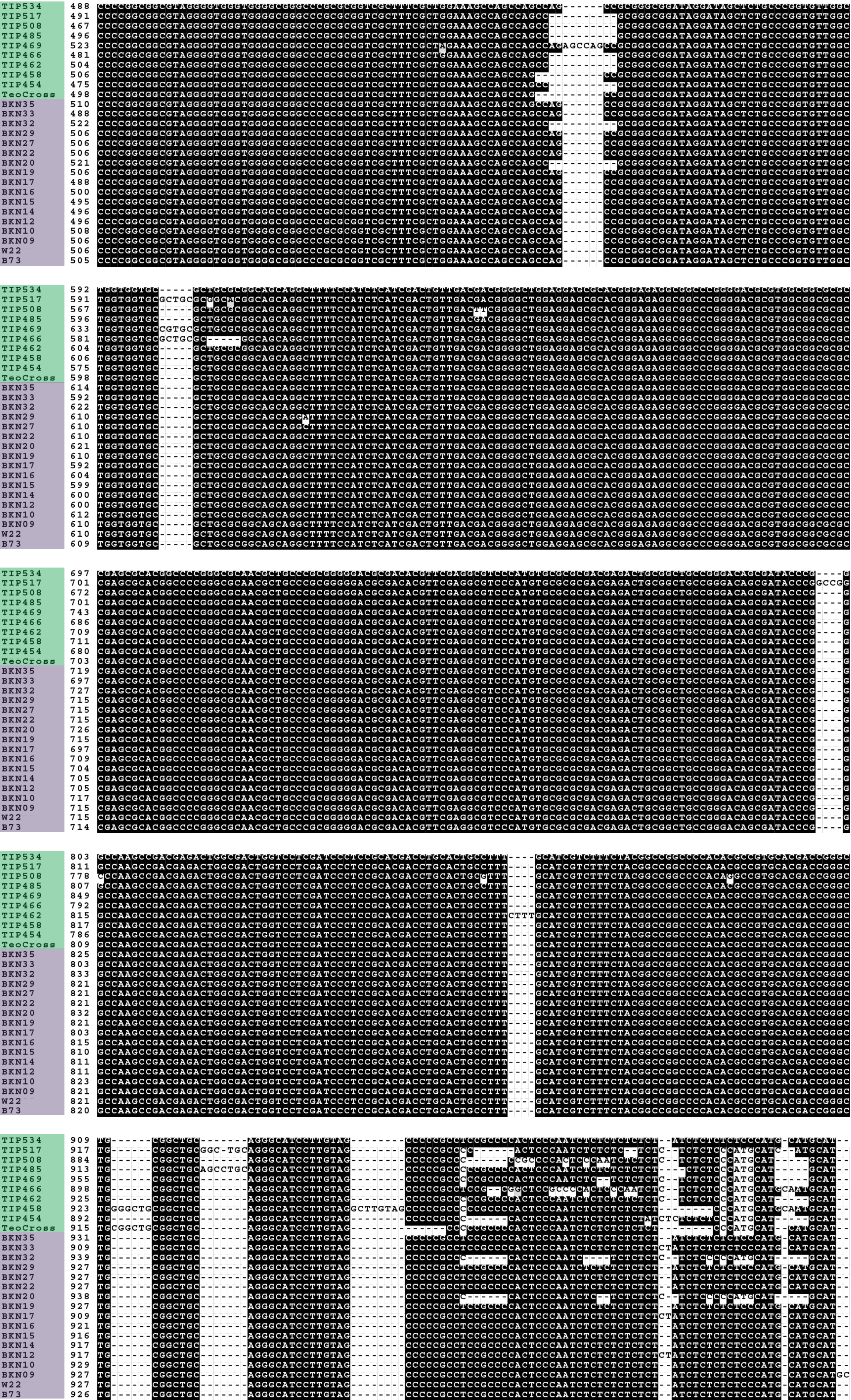

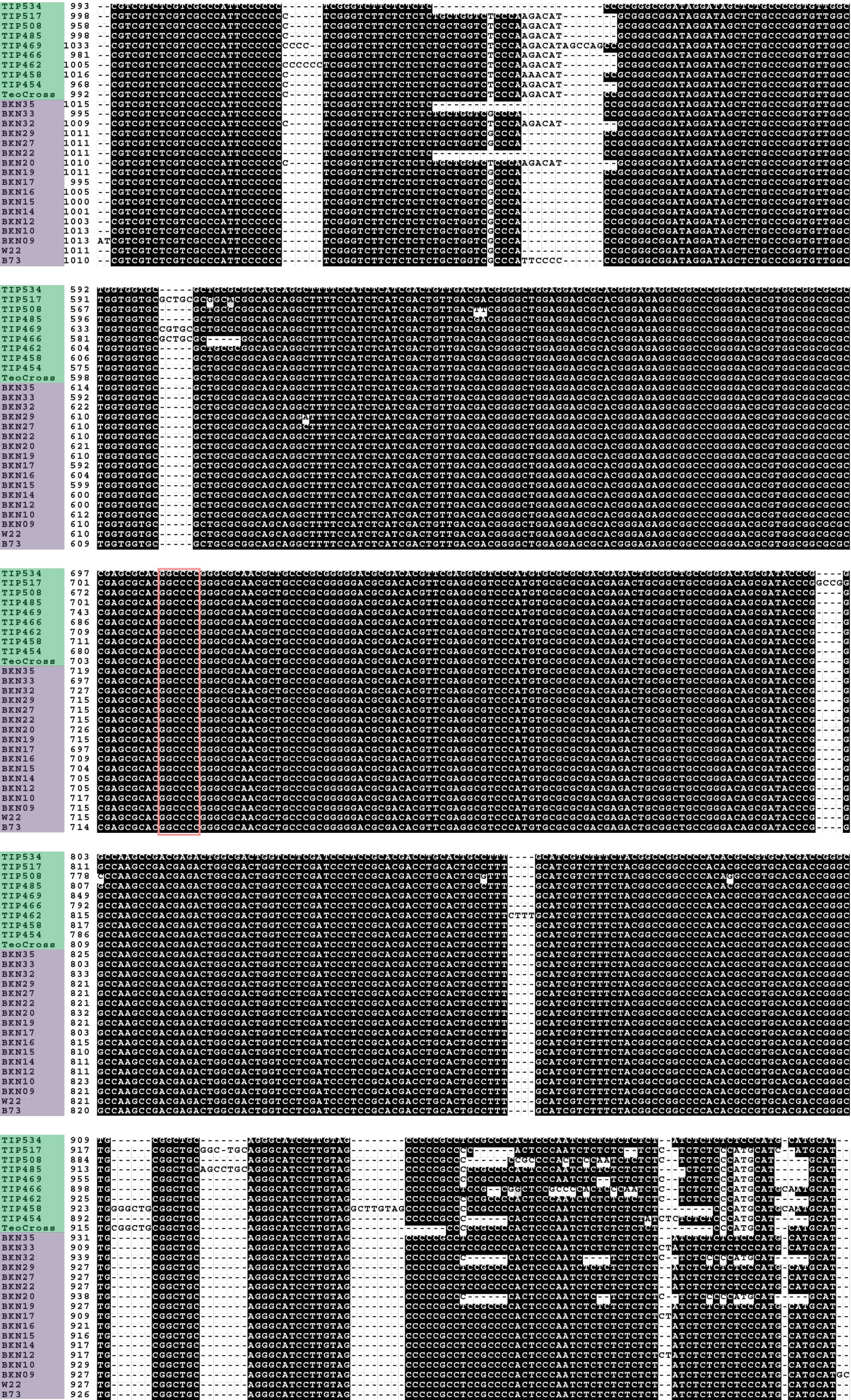

C

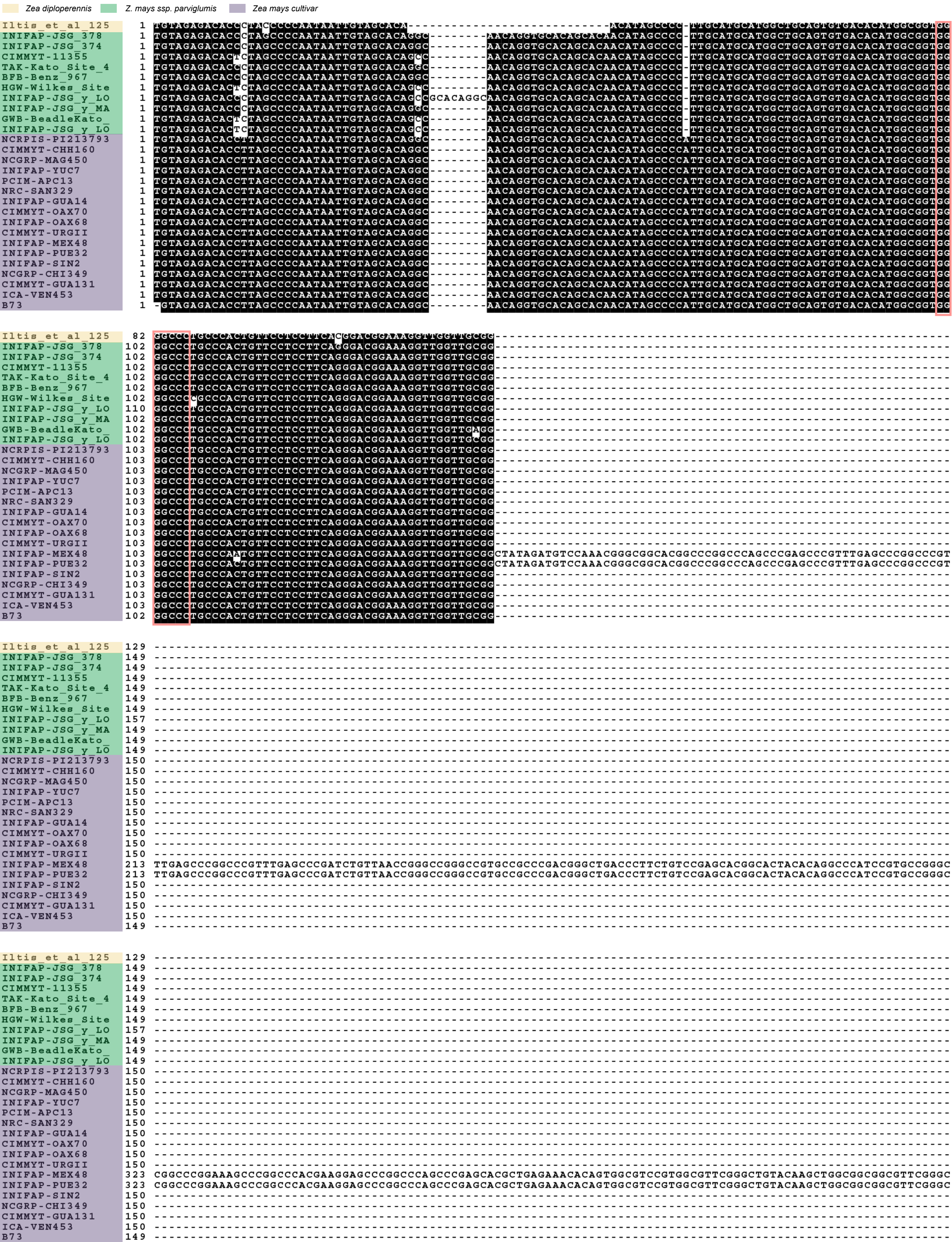
Zea diploperennis
Z. mays ssp. parviglumis
Zea mays cultivar

```
Iltis_et_al_125     129 --------------------------------------------------------------------------------------------------------------
INIFAP-JSG_378      149 --------------------------------------------------------------------------------------------------------------
INIFAP-JSG_374      149 --------------------------------------------------------------------------------------------------------------
CIMMYT-11355        149 --------------------------------------------------------------------------------------------------------------
TAK-Kato_Site_4     149 --------------------------------------------------------------------------------------------------------------
BFB-Benz_967        149 --------------------------------------------------------------------------------------------------------------
HGW-Wilkes_Site     149 --------------------------------------------------------------------------------------------------------------
INIFAP-JSG_y_LO     157 --------------------------------------------------------------------------------------------------------------
INIFAP-JSG_y_MA     149 --------------------------------------------------------------------------------------------------------------
GWB-BeadleKato      149 --------------------------------------------------------------------------------------------------------------
INIFAP-JSG_y_LO     149 --------------------------------------------------------------------------------------------------------------
NCRPIS-PI213793     150 --------------------------------------------------------------------------------------------------------------
CIMMYT-CHH160       150 --------------------------------------------------------------------------------------------------------------
NCGRP-MAG450        150 --------------------------------------------------------------------------------------------------------------
INIFAP-YUC7         150 --------------------------------------------------------------------------------------------------------------
PCIM-APC13          150 --------------------------------------------------------------------------------------------------------------
NRC-SAN329          150 --------------------------------------------------------------------------------------------------------------
INIFAP-GUA14        150 --------------------------------------------------------------------------------------------------------------
CIMMYT-OAX70        150 --------------------------------------------------------------------------------------------------------------
INIFAP-OAX68        150 --------------------------------------------------------------------------------------------------------------
CIMMYT-URGII        150 --------------------------------------------------------------------------------------------------------------
INIFAP-MEX48        433 TCTGGTGGGTGGTGCGCGTGTACGCCGTGCCGCTGCTGGTCGTCAACGCGTGG-CTGGTGCTCATCACGTGG-CTGGGACTAGCTGCGCGGCGCGCTCGCCACCGTCGAC
INIFAP-PUE32        433 TCTGGTGGGTGGTGCGCGTGTACGCCGTGCCGCTGCTGGTCGTCAACGCGTGGGCTGGTGCTCATCACGTGGGCTGGGACTAGCTGCGCGGCGCGCTCGCCACCGTCGAC
INIFAP-SIN2         150 --------------------------------------------------------------------------------------------------------------
NCGRP-CHI349        150 --------------------------------------------------------------------------------------------------------------
CIMMYT-GUA131       150 --------------------------------------------------------------------------------------------------------------
ICA-VEN453          150 --------------------------------------------------------------------------------------------------------------
B73                 149 --------------------------------------------------------------------------------------------------------------

Iltis_et_al_125     129 --------------------------------------------------------------------------------------------------------------
INIFAP-JSG_378      149 --------------------------------------------------------------------------------------------------------------
INIFAP-JSG_374      149 --------------------------------------------------------------------------------------------------------------
CIMMYT-11355        149 --------------------------------------------------------------------------------------------------------------
TAK-Kato_Site_4     149 --------------------------------------------------------------------------------------------------------------
BFB-Benz_967        149 --------------------------------------------------------------------------------------------------------------
HGW-Wilkes_Site     149 --------------------------------------------------------------------------------------------------------------
INIFAP-JSG_y_LO     157 --------------------------------------------------------------------------------------------------------------
INIFAP-JSG_y_MA     149 --------------------------------------------------------------------------------------------------------------
GWB-BeadleKato      149 --------------------------------------------------------------------------------------------------------------
INIFAP-JSG_y_LO     149 --------------------------------------------------------------------------------------------------------------
NCRPIS-PI213793     150 --------------------------------------------------------------------------------------------------------------
CIMMYT-CHH160       150 --------------------------------------------------------------------------------------------------------------
NCGRP-MAG450        150 --------------------------------------------------------------------------------------------------------------
INIFAP-YUC7         150 --------------------------------------------------------------------------------------------------------------
PCIM-APC13          150 --------------------------------------------------------------------------------------------------------------
NRC-SAN329          150 --------------------------------------------------------------------------------------------------------------
INIFAP-GUA14        150 --------------------------------------------------------------------------------------------------------------
CIMMYT-OAX70        150 --------------------------------------------------------------------------------------------------------------
INIFAP-OAX68        150 --------------------------------------------------------------------------------------------------------------
CIMMYT-URGII        150 --------------------------------------------------------------------------------------------------------------
INIFAP-MEX48        541 CGCGACTATGGCGTCC-TCAACCGCGTGTTCCACCACATCACGGACACGCACGTCACGCACCACCTCTTCACCTGTGAGGCTATGACTTATACGGGATTTGGGCTTGCGG
INIFAP-PUE32        543 CGCGACTATGGCGTCCCTCAACCGCGTGTTCCACCACATCACGGACACGTACGTCACGCACCACCTCTTCACCTGTGAGGCTATGACTTATACGGGATTTGGGCTTGCGG
INIFAP-SIN2         150 --------------------------------------------------------------------------------------------------------------
NCGRP-CHI349        150 --------------------------------------------------------------------------------------------------------------
CIMMYT-GUA131       150 --------------------------------------------------------------------------------------------------------------
ICA-VEN453          150 --------------------------------------------------------------------------------------------------------------
B73                 149 --------------------------------------------------------------------------------------------------------------

Iltis_et_al_125     129 --------------------------------------------------------------------------------------------------------------
INIFAP-JSG_378      149 --------------------------------------------------------------------------------------------------------------
INIFAP-JSG_374      149 --------------------------------------------------------------------------------------------------------------
CIMMYT-11355        149 --------------------------------------------------------------------------------------------------------------
TAK-Kato_Site_4     149 --------------------------------------------------------------------------------------------------------------
BFB-Benz_967        149 --------------------------------------------------------------------------------------------------------------
HGW-Wilkes_Site     149 --------------------------------------------------------------------------------------------------------------
INIFAP-JSG_y_LO     157 --------------------------------------------------------------------------------------------------------------
INIFAP-JSG_y_MA     149 --------------------------------------------------------------------------------------------------------------
GWB-BeadleKato      149 --------------------------------------------------------------------------------------------------------------
INIFAP-JSG_y_LO     149 --------------------------------------------------------------------------------------------------------------
NCRPIS-PI213793     150 --------------------------------------------------------------------------------------------------------------
CIMMYT-CHH160       150 --------------------------------------------------------------------------------------------------------------
NCGRP-MAG450        150 --------------------------------------------------------------------------------------------------------------
INIFAP-YUC7         150 --------------------------------------------------------------------------------------------------------------
PCIM-APC13          150 --------------------------------------------------------------------------------------------------------------
NRC-SAN329          150 --------------------------------------------------------------------------------------------------------------
INIFAP-GUA14        150 --------------------------------------------------------------------------------------------------------------
CIMMYT-OAX70        150 --------------------------------------------------------------------------------------------------------------
INIFAP-OAX68        150 --------------------------------------------------------------------------------------------------------------
CIMMYT-URGII        150 --------------------------------------------------------------------------------------------------------------
INIFAP-MEX48        650 CAGTAGTTGGGCCGTCCGCGTGCATAGCGAGCGTTAGCGTAAGCAGTCCAGGTGAATTTGTTGAACGGACCGGTTTAAAAGCCCGGCTGATAACGGGCCGTGCTCGGGCT
INIFAP-PUE32        653 CAGTAGTTGGGCCGTCCGCGTGCATAGCGAGCGTTAGCGTAAGCAGTCCAGGTGAATTTGTTGAACGGACCGGTTTAAAAGCCCGGCTGATAACGGGCCGTGCTCGGGCT
INIFAP-SIN2         150 --------------------------------------------------------------------------------------------------------------
NCGRP-CHI349        150 --------------------------------------------------------------------------------------------------------------
CIMMYT-GUA131       150 --------------------------------------------------------------------------------------------------------------
ICA-VEN453          150 --------------------------------------------------------------------------------------------------------------
B73                 149 --------------------------------------------------------------------------------------------------------------

Iltis_et_al_125     129 --------------------------------------------------------------------------------------------------------------
INIFAP-JSG_378      149 --------------------------------------------------------------------------------------------------------------
INIFAP-JSG_374      149 --------------------------------------------------------------------------------------------------------------
CIMMYT-11355        149 --------------------------------------------------------------------------------------------------------------
TAK-Kato_Site_4     149 --------------------------------------------------------------------------------------------------------------
BFB-Benz_967        149 --------------------------------------------------------------------------------------------------------------
HGW-Wilkes_Site     149 --------------------------------------------------------------------------------------------------------------
INIFAP-JSG_y_LO     157 --------------------------------------------------------------------------------------------------------------
INIFAP-JSG_y_MA     149 --------------------------------------------------------------------------------------------------------------
GWB-BeadleKato      149 --------------------------------------------------------------------------------------------------------------
INIFAP-JSG_y_LO     149 --------------------------------------------------------------------------------------------------------------
NCRPIS-PI213793     150 --------------------------------------------------------------------------------------------------------------
CIMMYT-CHH160       150 --------------------------------------------------------------------------------------------------------------
NCGRP-MAG450        150 --------------------------------------------------------------------------------------------------------------
INIFAP-YUC7         150 --------------------------------------------------------------------------------------------------------------
PCIM-APC13          150 --------------------------------------------------------------------------------------------------------------
NRC-SAN329          150 --------------------------------------------------------------------------------------------------------------
INIFAP-GUA14        150 --------------------------------------------------------------------------------------------------------------
CIMMYT-OAX70        150 --------------------------------------------------------------------------------------------------------------
INIFAP-OAX68        150 --------------------------------------------------------------------------------------------------------------
CIMMYT-URGII        150 --------------------------------------------------------------------------------------------------------------
INIFAP-MEX48        760 AGCCCAACGGGCTCAAATTCCTGTCGAGCCCGGCACGCTATCCGGGCCGGGCTAAGCCCGGGCCCGCTTCATTCCGTGCCGGGCCGGGCTCGTGTCGTGCTAAAAAATCG
INIFAP-PUE32        763 AGCCCAACGGGCTCAAATTCCTGTCGAGCCCGGCACGCTATCCGGGCCGGGCTAAGCCCGGGCCCGCTTCATTCCGTGCCGGGCCGGGCTCGTGTCGTGCTAAAAAATCG
INIFAP-SIN2         150 --------------------------------------------------------------------------------------------------------------
NCGRP-CHI349        150 --------------------------------------------------------------------------------------------------------------
CIMMYT-GUA131       150 --------------------------------------------------------------------------------------------------------------
ICA-VEN453          150 --------------------------------------------------------------------------------------------------------------
B73                 149 --------------------------------------------------------------------------------------------------------------
```

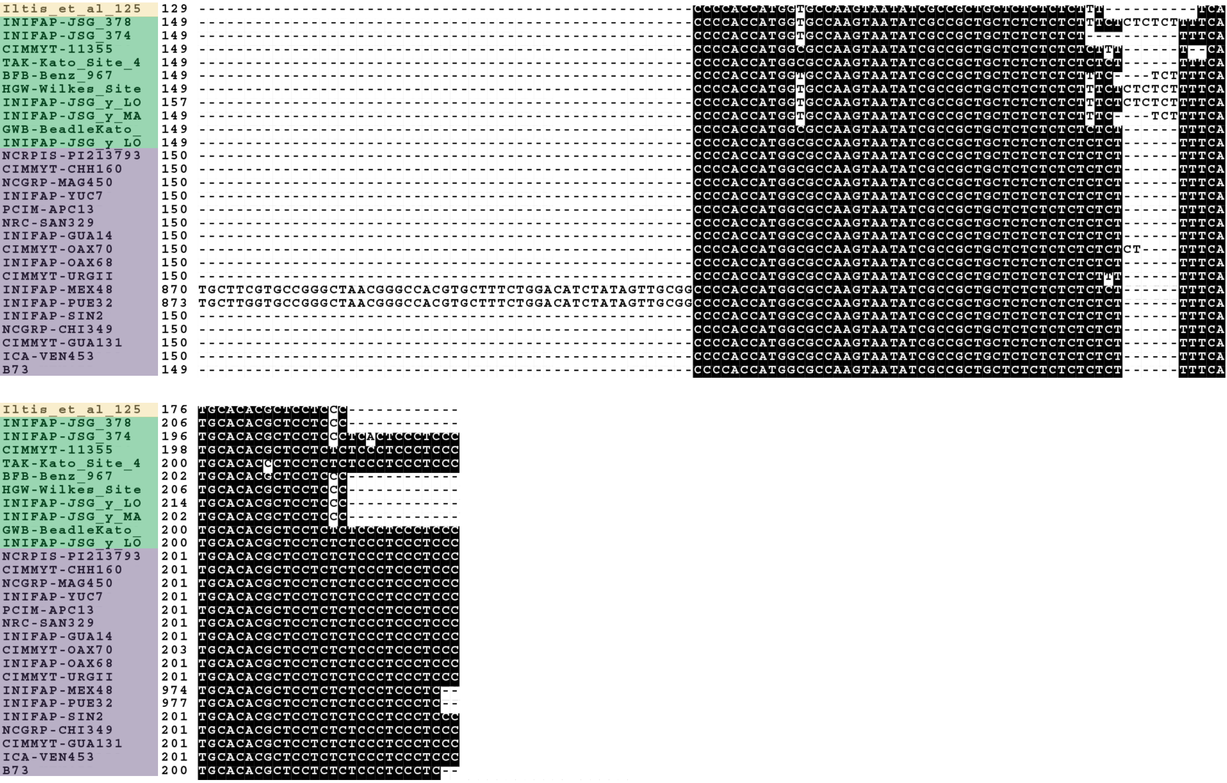

Supplementary Data 17. Sequence alignment of the TB1 binding region in the *tb1 Hopscotch* region (A), prolificacy locus (B) and *tga1* promoter (C). Pink square frames represent the GGNCCC TB1 binding motif at the summit of each peak. The sequence of *Zea mays* landraces, *Zea mays ssp. parviglumis* and *Zea mays ssp. mexicana* in (A) are downloaded from Studer *et al.*, 2011; the sequence of *Zea mays* cultivars and *Zea mays ssp. parviglumis* in (B) are downloaded from Wills *et al.*, 2013; and the sequence of *Zea diploperennis*, *Z. mays ssp. parviglumis* and *Zea mays* cultivars in (C) are downloaded from Wang *et al.*, 2005.
