# Supplementary material for: The regulatory landscape of a core maize domestication module controlling bud dormancy and growth repression
Source: Nat Commun. 2019 Aug 23;10:3810. doi: 10.1038/s41467-019-11774-w (PMC6707278; doi:10.1038/s41467-019-11774-w)
Supplement: Supplementary file 21 — Reporting Summary [file 41467_2019_11774_MOESM21_ESM.pdf]

naturereresearch

Corresponding author(s):

Last updated by author(s):

✘

✘

✘

✘

✘

✘

✘

✘

✘

✘

Illumina Hi-seq 2500 sequencer and Illumina Hi-seq 4000 platform

FastQC, Trimmomatic v.0.36, STAR aligner v.2.6.0a, R package edgeR v.3.22.5, AgriGO v2.0, ggplot2, Bowtie2, MACS2, MEME

Provide your data availability statement here.

✘
